# Real-world effectiveness of Paxlovid in protecting Long COVID for outpatient adult patients: A target trial emulation study from the RECOVER Initiative

**DOI:** 10.1371/journal.pgph.0005771

**Published:** 2026-10-28

**Authors:** Chengxi Zang, Haoyang Li, Dhruv Khullar, Yongkang Zhang, Stephenson Strobel, Yong Chen, Marc A. Sala, Payal Bharat Patel, Alejandro P. Comellas, Andrew Wylam, Mark Weiner, Chris B. Forrest, Thomas Carton, Fei Wang, Rainu Kaushal, on behalf of the RECOVER Consortium

**Affiliations:** 1 Department of Population Health Sciences, Weill Cornell Medicine, New York, New York, United States of America; 2 Department of Biostatistics, Epidemiology, and Informatics, University of Pennsylvania Perelman School of Medicine, Philadelphia, Pennsylvania, United States of America; 3 Division of Pulmonary and Critical Care, Northwestern University Feinberg School of Medicine, Chicago, Illinois, United States of America; 4 Department of Neurology, University of Washington School of Medicine, Seattle, Washington, United States of America; 5 Division of Pulmonary, Critical Care and Occupational Medicine, University of Iowa, Iowa City, Iowa, United States of America; 6 Patient representative for Long COVID patients with the National Institutes of Health’s RECOVER Initiative, Bethesda, Maryland, United States of America; 7 Applied Clinical Research Center, Children’s Hospital of Philadelphia, Philadelphia, Pennsylvania, United States of America; 8 Louisiana Public Health Institute, New Orleans, Louisiana, United States of America; PLOS: Public Library of Science, UNITED STATES OF AMERICA

## Abstract

Paxlovid (nirmatrelvir/ritonavir) has been approved for use in patients who are at high risk for severe acute COVID-19 illness. Evidence regarding whether acute use of Paxlovid protects against Post-Acute Sequelae of SARS-CoV-2 infection (PASC), or Long COVID, is mixed in high-risk patients and lacking in low-risk patients. With a target trial emulation framework, we evaluated the association of Paxlovid treatment within 5 days of SARS-CoV-2 infection with incident Long COVID and hospitalization or death from any cause in the post-acute period (30–180 days after infection) using electronic health records from the Patient-Centered Clinical Research Networks (PCORnet) RECOVER repository. The study population included 497,499 SARS-CoV-2 positive patients between March 1, 2022, to February 28, 2023. For those who had at least 1 risk factor for severe COVID-19 illness, 159,659 were treated with at least one dose of Paxlovid within 5 days since infection and 272,048 were not treated with Paxlovid or other COVID-19 treatments during the acute infection period. Compared with the non-treated group, Paxlovid treatment was associated with reduced risk of Long COVID with a Hazard Ratio (HR) of 0.88 (95% CI, 0.87 to 0.89) and absolute risk reduction of 2.99 events per 100 persons (95% CI, 2.65 to 3.32). For those without documented risk factors, the associations (HR, 1.03, 95% CI 0.95 to 1.11; risk increase 0.80 events per 100 persons, 95% CI -0.84 to 2.45) were inconclusive. High-risk, nonhospitalized adult patients with COVID-19 who were treated with Paxlovid within 5 days of SARS-CoV-2 infection had a lower risk of Long COVID and all-cause hospitalization or death in the post-acute period. However, Long COVID risk reduction with Paxlovid was not observed in low-risk patients.

## Introduction

Some patients who recover from the acute phase of COVID-19 infection develop new, prolonged, or exacerbated medical conditions, often referred to as Post-Acute Sequalae of SARS-CoV-2 Infection (PASC) or Long COVID [1–7]. No standard definition or diagnostic criteria exists for Long COVID, and researchers are still clarifying the biological mechanism through which these conditions develop [4].

Although no treatments for Long COVID have been approved by the U.S. Food and Drug Administration, there is growing interest in whether the antiviral, Paxlovid (or nirmatrelvir/ritonavir), could mitigate the long-term consequences of SARS-CoV-2 infection. Paxlovid received emergency use authorization (EUA) in December 2021 for treatment of non-hospitalized COVID-19 patients with mild to moderate acute infection who are at high risk of progressing to severe COVID-19 (treatment recommended within 5 days of symptom onset) [8,9]; millions of people in the US have since received Paxlovid for acute COVID-19. Although studies have shown that Paxlovid is effective at reducing the risk of severe COVID-19, including hospitalization or death [9], limited research exists on whether it is effective in preventing Long COVID, and existing studies have found mixed results [10–12]. Further, the association of Paxlovid with Long COVID risk is largely lacking in low-risk patients. A few low-risk patients were prescribed Paxlovid for acute illness [2], also providing the opportunity for examining the effectiveness of reducing Long COVID risk in those low-risk patients.

To date, the largest observational retrospective study on Paxlovid as a preventative treatment for Long COVID was conducted through an analysis of electronic health records (EHR) from 281,793 individuals with SARS-CoV-2 infection and at least 1 risk factor for progression to severe COVID-19 in the Veteran Affairs (VA) system [13]. The study found that receipt of Paxlovid was associated with a reduced risk of Long COVID (hazard ratio 0.74; 95% CI, 0.72-0.77; absolute risk reduction, 4.51%; 95% CI, 4.01-4.99), as well as post-acute death or hospitalization [13]. However, the study population, which was older and overwhelmingly white and male, was not representative of the general U.S. population, and the effectiveness of Paxlovid in preventing Long COVID remains unclear in demographically diverse populations.

In this study, we analysed data from 29 EHR repositories of the national Patient-Centered Clinical Research Networks (PCORnet) as part of the NIH RECOVER initiative. We studied the association between Paxlovid treatment within 5 days of acute COVID-19 and the development of Long COVID, all-cause hospitalization, and all-cause mortality in the post-acute phase. We followed the target trial emulation framework [9,14,15], together with the large observational cohort, aiming to examine the associations of Paxlovid and the risk of Long COVID in general population.

## Methods

### Data

The PCORNet RECOVER Database. The PCORnet RECOVER infrastructure leveraged PCORnet to develop a single, unified EHR/RWD repository to study PASC across ~28.25 million (18.75 million adult - 9.5 million paediatric) patients from 40 adult and paediatric health systems nationwide who continue to refresh their data at least quarterly. The source data includes patients tested for COVID-19 (regardless of result), those diagnosed with COVID-19, those who received COVID-19 vaccine and therapeutics (e.g., Remdesivir and Paxlovid), and/or those who have received a respiratory diagnosis since 2019. The enclave contains structured EHR data consisting of inpatient and ambulatory encounters, laboratory results, vital signs, medications, diagnoses, procedures, birthdates, gender, and race information. The EHR data is linked to geocoded data to the level of the census tract, block group, and/or 9-digit zip code to allow linkage to exposome information to assess the influence of Social Determinants of Health (SDoH) and environmental exposures on COVID-19 outcomes. In addition, our data enclave includes clinical notes for Natural Language Processing (NLP), vaccine registries, and death registries.

### Study cohort

This observational analysis emulated a target trial [9,14] to determine whether Paxlovid treatment for acute COVID-19 reduces the risk of Long COVID among nonhospitalized adults with COVID-19. We used data from 29 EHR repositories of the national Patient-Centered Clinical Research Network (PCORnet) as part of the NIH RECOVER initiative. Patients were identified as having acute COVID-19 if they had (a) positive SARS-CoV-2 polymerase-chain-reaction (PCR) or antigen laboratory tests; (b) the International Classification of Diseases, Tenth Revision, Clinical Modification (ICD-10-CM) diagnosis code U07.1 representing COVIID-19 diagnosis; or (c) Paxlovid (nirmatrelvir/ritonavir) or Remdesivir prescriptions. The protocol is summarized in Table A in S1 Appendix.

Key eligibility criteria included age 18 years or greater at the first confirmed SARS-CoV-2 infection between March 1^st^, 2022 and February 28, 2023, interactions with the healthcare system in the past 3 years to 7 days before the index event, risk factors for progression to severe COVID-19 illness (≥50 years or having at least 1 underlying medical conditions associated with an increased risk of developing severe illness from COVID-19), no previous ICD-10-CM diagnosis codes of U0.99 (Post COVID-19 condition, unspecified) or B94.8 (Sequelae of other specified infectious and parasitic diseases), nonhospitalization for the treatment of COVID-19, and absence of severe liver disease, dialysis or severe renal impairment, use of other COVID-19 treatments including antiviral, antibody, or convalescent plasma treatments, or contraindication for Paxlovid. See Table A in S1 Appendix for the detailed specifications of protocols of the target trial and Table B in S1 Appendix for the study variables and associated time windows.

### Exposure

The exposure of interest was receipt of Paxlovid (nirmatrelvir/ritonavir) within 5 days after the SARS-CoV-2 infection. Patients were assigned to the treatment group if they had a documented prescription for Paxlovid within this time window. The control group consisted of patients who did not receive Paxlovid or other COVID-19 treatments during the acute infection period. Because treatment was not randomly assigned in routine clinical care, we emulated randomization by adjusting for a comprehensive set of baseline covariates using stabilized inverse probability of treatment weighting (IPTW) based on propensity scores. The index date for treated group was defined as the date of the first Paxlovid prescription and the index date for the control group was defined as the date of the first documented evidence of SARS-CoV-2 infection.

### Outcomes

The outcomes of interest were incident post-acute sequelae of SARS-CoV-2 (PASC, or Long COVID), all-cause death, and all-cause hospitalization in the post-acute phase. The Long COVID definition is a rules-based computable phenotype leveraging ICD codes for 24 condition clusters [6,7]. See the detailed condition clusters in S1 Appendix-Long COVID definition section. Follow-up began 30 days after the SARS-CoV-2 infection. Patients were followed until the earliest occurrence of the incident outcome of interest in the post-acute phase, death, 180 days after SARS-CoV-2 infection, or the end of the study period (August 1, 2023), whichever occurred first. These events defined the censoring mechanisms in the survival analyses. The death event, both in acute and post-acute phases, was treated as a competing event that precludes other outcomes of interest from occurring.

### Covariates

A broad range of potential confounders was considered, including sex, age, race and ethnicity, the Area Deprivation Index (ADI), body mass index (BMI), time of infection, smoking status, COVID-19 vaccine status, baseline clinical risk factors (cancer, chronic kidney disease, chronic liver disease, chronic lung diseases, cystic fibrosis, dementia or other neurological conditions, diabetes (type 1 or type 2), disabilities, heart conditions, HIV infection, immunocompromised condition or weakened immune system, mental health conditions, overweight and obesity, pregnancy, sickle cell disease or anemia, smoking, stroke or cerebrovascular disease, substance use disorders, tuberculosis) [16] for developing severe COVID-19 illness, and additional covariates (coagulopathy, peripheral vascular disorders, seizure/epilepsy, weight loss, obstructive sleep apnea, Epstein-Barr and Infectious Mononucleosis, Herpes Zoster) might be associated with treatment and PASC outcomes [6,7,17,18]. Covariates and assessment periods are summarized in Table B in S1 Appendix.

### Statistical analysis

Baseline covariate balance was compared between the two exposure groups by standardized mean differences (SMD) both before and after applying stabilized inverse probability of treatment weighting (IPTW), with a difference of less than 0.1 considered to be balanced. The stabilized inverse probability of treatment weighting (IPTW) was used in the survival analysis to adjust for the baseline covariates [19,20]. The propensity scores for different exposures were calculated with the regularized logistic regression with L2 norm, with exposure to Paxlovid or not as the dependent variable, and with all the baseline covariates as independent variables [7,15]. Cumulative incidence curves were estimated using the Aalen–Johansen estimator in the reweighted population, treating death as a competing risk for non-mortality outcomes. The hazard ratios were estimated by the Cox survival model in the reweighted population, and two-sided 95% confidence intervals were calculated with the use of a robust variance estimator to account for stabilized IPTW weights. The absolute risk reduction was the difference in cumulative incidences at 180 days of follow-up between exposed and non-exposed groups. The nonparametric 1000-iteration bootstrapping estimation of risks, risk reductions, and percentile-based 95% confidence intervals were also calculated. In each bootstrap iteration, individuals were resampled with replacement, propensity scores were re-estimated using the same model specification, stabilized inverse probability of treatment weights were recalculated, and the weighted Cox models were refitted to obtain the bootstrap estimates. The missing values in baseline covariates were treated as separate categories for categorical variables when appropriate or handled according to standard PCORnet data conventions as shown in Table 1 and Table B in S1 Appendix.

**Table 1:** Population characteristics of adult SARS-CoV-2 infected patients for evaluating the effectiveness of the acute prescription of Paxlovid on the PASC conditions, during the period from March 1, 2022, to February 28, 2023.

	Paxlovid treated SARS-CoV-2 Infected Patients. (N = 165,256)	Control group (N = 307,922)	SMD
**Characteristic**			
**Sex — no. (%)**			
Female	100,931 (61.1)	196,527 (63.8)	-0.06
Male	64,300 (38.9)	111,224 (36.1)	0.06
Other/Missing	25 (0.0)	171 (0.1)	-0.02
**Median age (IQR) — yr**	61 (48—71)	50 (35—65)	0.49
**Age group — no. (%)**			
18-24	3,649 (2.2)	26,292 (8.5)	-0.28
25-34	12,128 (7.3)	53,258 (17.3)	-0.31
35-49	30,936 (18.7)	74,274 (24.1)	-0.13
50-64	50,522 (30.6)	74,914 (24.3)	0.14
65+	68,021 (41.2)	79,184 (25.7)	0.33
**Race/Ethnicity — no. (%)**			
Asian Non-Hispanic	6,453 (3.9)	17,258 (5.6)	-0.08
Black or African American Non-Hispanic	15,125 (9.2)	43,307 (14.1)	-0.15
Hispanic or Latino Any Race	9,848 (6.0)	37,630 (12.2)	-0.22
White Non-Hispanic	116,020 (70.2)	175,712 (57.1)	0.28
Other Non-Hispanic	3,545 (2.1)	9,772 (3.2)	-0.06
Unknown	14,265 (8.6)	24,243 (7.9)	0.03
**No. of hospital visits in the past 3 yr — no. (%)**			
Inpatient 0	138,148 (83.6)	261,619 (85.0)	-0.04
Inpatient 1–2	19,765 (12.0)	35,296 (11.5)	0.02
Inpatient >=3	7,343 (4.4)	11,007 (3.6)	0.04
Outpatient 0	1,544 (0.9)	11,394 (3.7)	-0.18
Outpatient 1–2	7,899 (4.8)	26,481 (8.6)	-0.15
Outpatient >=3	155,813 (94.3)	270,047 (87.7)	0.23
Emergency 0	130,061 (78.7)	223,574 (72.6)	0.14
Emergency 1–2	27,353 (16.6)	58,644 (19.0)	-0.07
Emergency >=3	7,842 (4.7)	25,704 (8.3)	-0.15
**Median Area Deprivation Index (IQR) — rank**	30 (12—54)	37 (16—61)	-0.20
**BMI (IQR)**	29 (25—35)	29 (24—35)	0.03
**Documented Vaccination Status**			
Fully vaccinated	67,718 (41.0)	111,921 (36.3)	0.10
Partially vaccinated	26,333 (15.9)	41,717 (13.5)	0.07
No evidence	72,282 (43.7)	156,552 (50.8)	-0.14
**Patients in different Index time — no. (%)**			
03/22-06/22	48,541 (29.4)	117,644 (38.2)	-0.19
07/22-10/22	68,535 (41.5)	127,243 (41.3)	0.00
11/22-02/23	48,180 (29.2)	63,035 (20.5)	0.20
**Patient w/o documented risks— no. (%)**	5,597 (3.4)	35,874 (11.7)	-0.32
**Pregnant— no. (%)**	822 (0.5)	4,368 (1.4)	-0.09
**Patient with at least one risk factor— no. (%)**	158,837 (96.1)	267,680 (86.9)	0.33
**Individual Risk Factors — no. (%)**			
Cancer	20,016 (12.1)	23,277 (7.6)	0.15
Chronic kidney disease	10,709 (6.5)	20,746 (6.7)	-0.01
Chronic liver disease	11,232 (6.8)	17,398 (5.7)	0.05
Chronic lung disease	33,140 (20.1)	55,918 (18.2)	0.05
Cystic fibrosis	224 (0.1)	225 (0.1)	0.02
Dementia or other neurological conditions	4,897 (3.0)	7,272 (2.4)	0.04
Diabetes	25,877 (15.7)	41,410 (13.4)	0.06
Disabilities	1,340 (0.8)	3,045 (1.0)	-0.02
Heart conditions	33,471 (20.3)	50,986 (16.6)	0.10
Hypertension	68,942 (41.7)	101,995 (33.1)	0.18
HIV infection	1,173 (0.7)	1,839 (0.6)	0.01
Immune dysfunction	62,773 (38.0)	81,236 (26.4)	0.25
Mental health conditions	27,472 (16.6)	56,395 (18.3)	-0.04
Overweight and obesity	108,679 (65.8)	185,509 (60.2)	0.11
Sickle cell disease or thalassemia	18,546 (11.2)	34,254 (11.1)	0.00
Smoking current or former	32,814 (19.9)	59,393 (19.3)	0.01
Stroke or cerebrovascular disease	9,363 (5.7)	14,048 (4.6)	0.05
Substance use disorders	11,894 (7.2)	33,630 (10.9)	-0.13
Tuberculosis	77 (0.0)	199 (0.1)	-0.01

a. The SARS-CoV-2 positive patients were identified by a) positive SARS-CoV-2 polymerase-chain-reaction (PCR) or antigen laboratory tests; b) the ICD-10-CMdiagnosis code U07.1 representing COVIID-19 diagnosis; or c) Paxlovid (nirmatrelvir/ritonavir) or Remdesivir prescriptions. The first documented evidence was defined as the index event. IQR denotes the interquartile range. The percentage may not sum up to 100 because of rounding. ADI, the Area Deprivation Index. BMI, Body Mass Index. b. A standardized mean difference (SMD) of >0.10 or <-0.10 indicates an important effect size difference between the two samples, otherwise, no significant difference is assumed. c. Pregnant patients were included when SARS-CoV-2 infection occurred during pregnancy.

We conducted subgroup analyses according to sex (female or male), race (Black or White), age (<65 or ≥ 65 years), different baseline risk factors, infection time, and areas with different rural-urban commuting patterns. For subgroup analyses, infection time was grouped into two broader periods separated by October 1, 2022 to approximate different waves of potentially different variants of concerns. In addition to investigating the at-risk patients, we also investigated patients without documented risk factors. Multiple sensitivity analyses were conducted to test the robustness of the results, including using a different Long COVID definition with a focus on fatigue, cognitive, and respiratory symptom clusters [21], cohorts similar to the US Veterans study (87.8% male patients aged ≥ 60 years old or having any risk factors) [13]. We further conducted two negative outcome control studies to explore the possibility of residual confounding [22]. Negative control outcomes were selected based on the absence of a plausible causal relationship with Paxlovid treatment and but share the same potential confounders as the primary outcomes. These outcomes were identified based on clinical knowledge and prior literature (e.g., benign neoplasms, skin cancer, etc as shown in Figs C and D in S1 Appendix). If the estimated associations between treatment and these negative control outcomes were close to the null, this would provide evidence supporting the validity of the study design.

### Ethics

This study received Biomedical Research Alliance of New York (BRANY) institutional review board (IRB) approval #21-08-508. As part of the Biomedical Research Alliance of New York (BRANY IRB) process, the protocol has been reviewed in accordance with the institutional guidelines. The Biomedical Research Alliance of New York (BRANY) waived the need for consent and HIPAA authorization.

## Results

### Characteristics of the study population

Among 558,037 patients with their first SARS-CoV-2 infection between March 1, 2022, and February 28, 2023, 497,499 were eligible for the study (Fig 1). Among eligible adult patients, 165,256 received Paxlovid within 5 days of infection and 307,922 did not. The baseline characteristics of the two groups are presented in Table 1.

**Fig 1:**
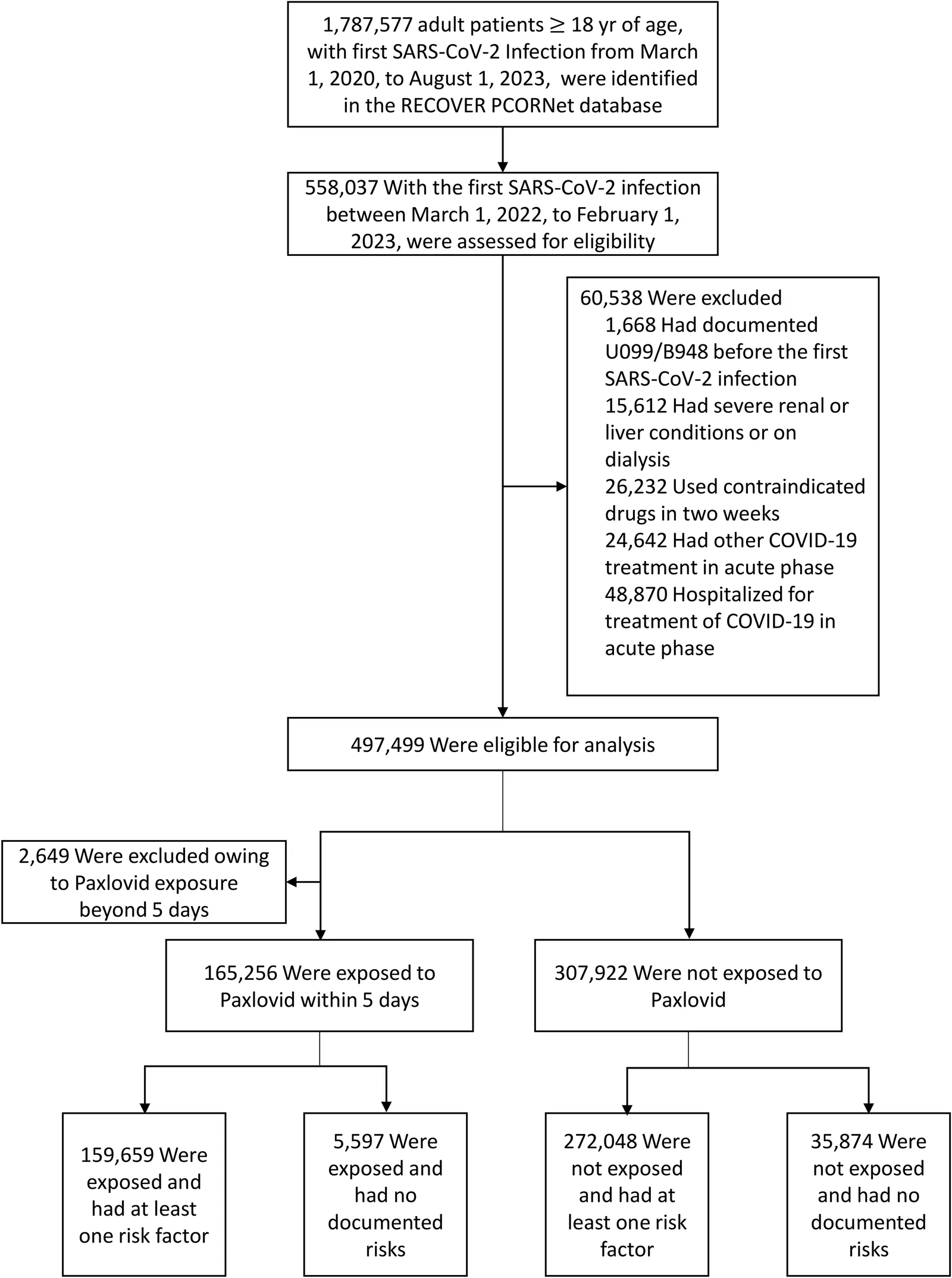
Selection of adult SARS-CoV-2 infected patients for evaluating the effectiveness of the acute prescription of Paxlovid on the PASC conditions, during the period from March 1, 2022, to February 28, 2023.

The median age in the Paxlovid-treated group was older than the non-treated group (61 [interquartile range (IQR), 48–71 vs 50 [IQR 35–65]). Paxlovid-treated patients were more likely to be (self-reported) White than non-treated patients, and more likely to have a risk factor (e.g., cancer, hypertension, immune dysfunction, overweight, and obesity) for developing severe COVID-19 illness. They were less likely to be (self-reported) Black or Hispanic, or have substance use disorders.

Comparisons were conducted for the Paxlovid-treated group and non-treated group and all measured variables were well-balanced between corresponding comparisons as summarized in Figs A and B in S1 Appendix. In addition, significant associations of exposure were not observed with negative outcomes, as summarized in Figs C and D in S1 Appendix, suggesting little residual confounding.

### Paxlovid effectiveness

At 180 days of follow-up, the estimated risk of Long COVID was 30.51 events per 100 persons (95% confidence interval (CI), 30.25 to 30.77) in the Paxlovid-treated group and 33.50 (95% CI, 33.28 to 33.71) in the non-treated group (Fig 2). Compared to patients in the non-treatment group, Paxlovid-treated patients had a reduced risk of Long COVID, with a Hazard Ratio (HR) of 0.88 (95% CI, 0.87 to 0.89) and risk reduction of 2.99 events per 100 persons (95% CI, 2.65 to 3.32). Paxlovid treatment was also associated with a lower risk of all-cause hospitalization (HR, 0.70, 95% CI, 0.68-0.73; risk reduction of 2.37 events per 100 persons, 95% CI, 2.19 to 2.56), and all-cause death (HR, 0.53, 95% CI, 0.46 to 0.60; risk reduction of 0.23 events per 100 persons, 95% CI, 0.19 to 0.28) in the post-acute 180 days.

**Fig 2:**
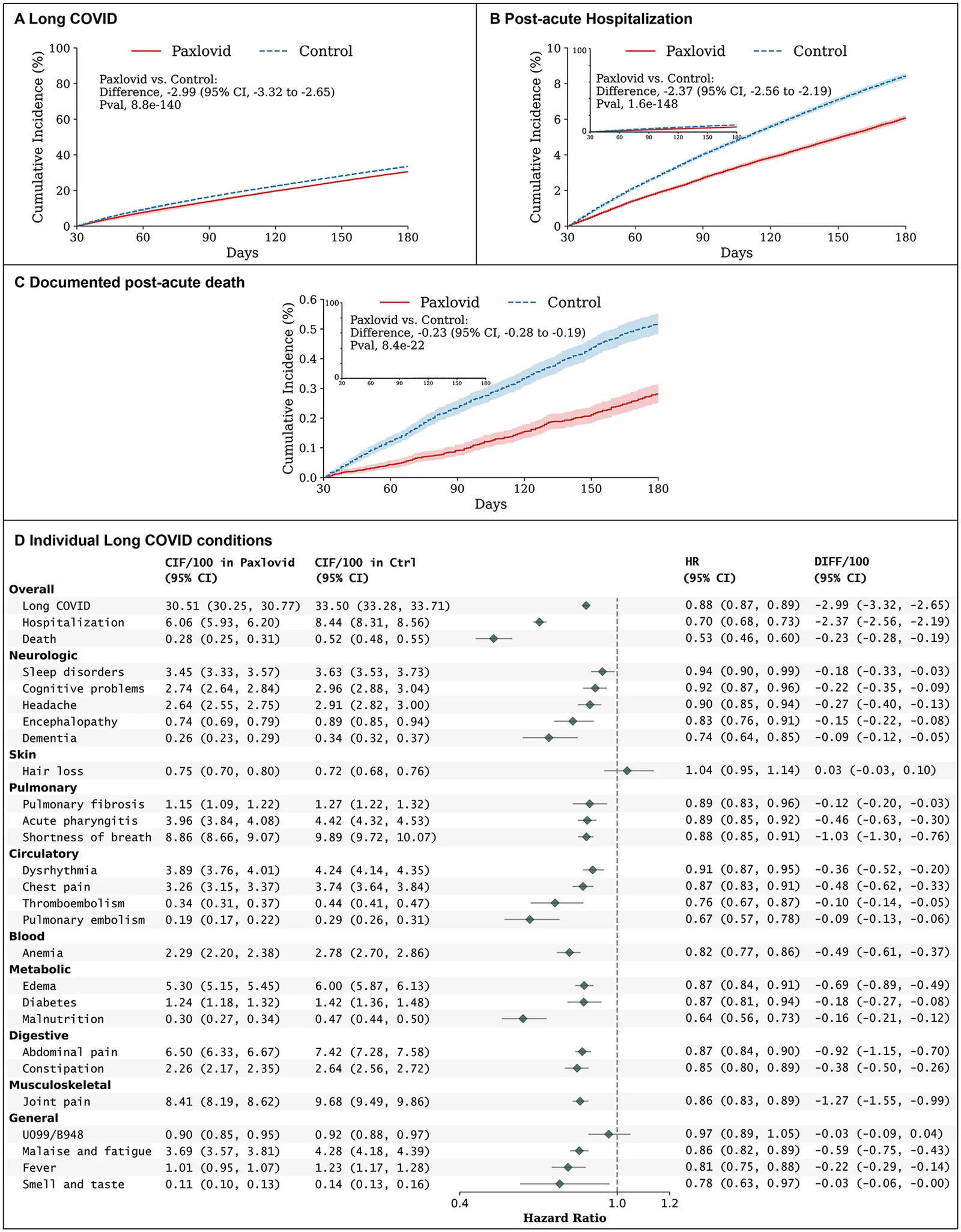
Absolute risk, risk ratio, and risk difference of long COVID in the Paxlovid group versus the Non-treated group among high-risk patients. A, the cumulative incidence of Long COVID; B, the cumulative incidence of post-acute death; C, the cumulative incidence of post-acute hospitalization; and D, the absolute risk, risk ratio, and risk difference of individual Long COVID at 180 days after the infection. Outcomes were ascertained 30 days after the first documented SARS-CoV-2 infection evidence until the end of the follow-up. The absolute risk, risk ratio, and risk difference were captured by the cumulative incidence (CIF), hazard ratio (HR), and the difference of cumulative incidence per 100 persons (DIFF/100), estimated at 180 days after the infection date, respectively. Shaded areas represent pointwise 95% Confidence Intervals (95% CI).

Paxlovid treatment was associated with a lower risk of incident Long COVID across systems as shown in Fig 2D, including post-acute neurological conditions (sleep disorders, cognitive problems, headache, encephalopathy, dementia), post-acute pulmonary conditions (pulmonary fibrosis, acute pharyngitis, shortness of breath), post-acute blood conditions (anemia), post-acute metabolic conditions (edema, diabetes, malnutrition), post-acute digestive conditions (abdominal pain, constipation), post-acute musculoskeletal conditions (joint pain), and some general conditions in the post-acute phase (e.g., malaise and fatigue, fever, smell, and taste). There were no conclusive associations between Paxlovid and hair loss (HR, 1.04, 95% CI 0.95 to 1.14), or ICD-10 codes U0.99 or B94.8 representing unspecified Post COVID-19 conditions or sequelae of infectious illness (HR, 0.97, 95% CI 0.89 to 1.05).

Paxlovid treatment was also associated with a lower risk of incident Long COVID in different subpopulations stratified by sex, race, age, baseline risk conditions (except for pregnant females), infection time, and different rural-urban commuting areas as shown in Fig 3. Specifically, the weighted Cox proportional hazards models were fitted separately within each predefined subgroup. Relatively greater risk reductions were observed in males (3.53 events per 100 persons, 95% CI, 2.98 to 4.08) compared to females (2.61, 95% 2.18 to 3.04) and for individuals aged ≥ 65 (3.27, 95% CI, 2.74 to 3.81) compared to those under 65 (2.73, 95% CI, 2.29 to 3.17). Regarding the rural and urban areas, the greatest risk reduction was found in small towns (6.90 events per 100 persons, 95% CI, 4.28 to 9.52), followed by micropolitan (5.69, 95% CI, 3.96 to 7.42) and metropolitan patients (2.19, 95% CI, 1.83 to 2.56). The subgroup analyses for post-acute all-cause hospitalization or death are in Fig F and G in S1 Appendix. Results were broadly similar in sensitivity analyses that considered a secondary Long COVID definition with a focus on cognitive, fatigue, and respiratory conditions, and in a population with demographics similar to the Veteran Affairs study (87.8% male patients aged ≥ 60 years old or having any risk factors) (Fig 3 and Fig H in S1 Appendix for the subgroup analyses).

**Fig 3:**
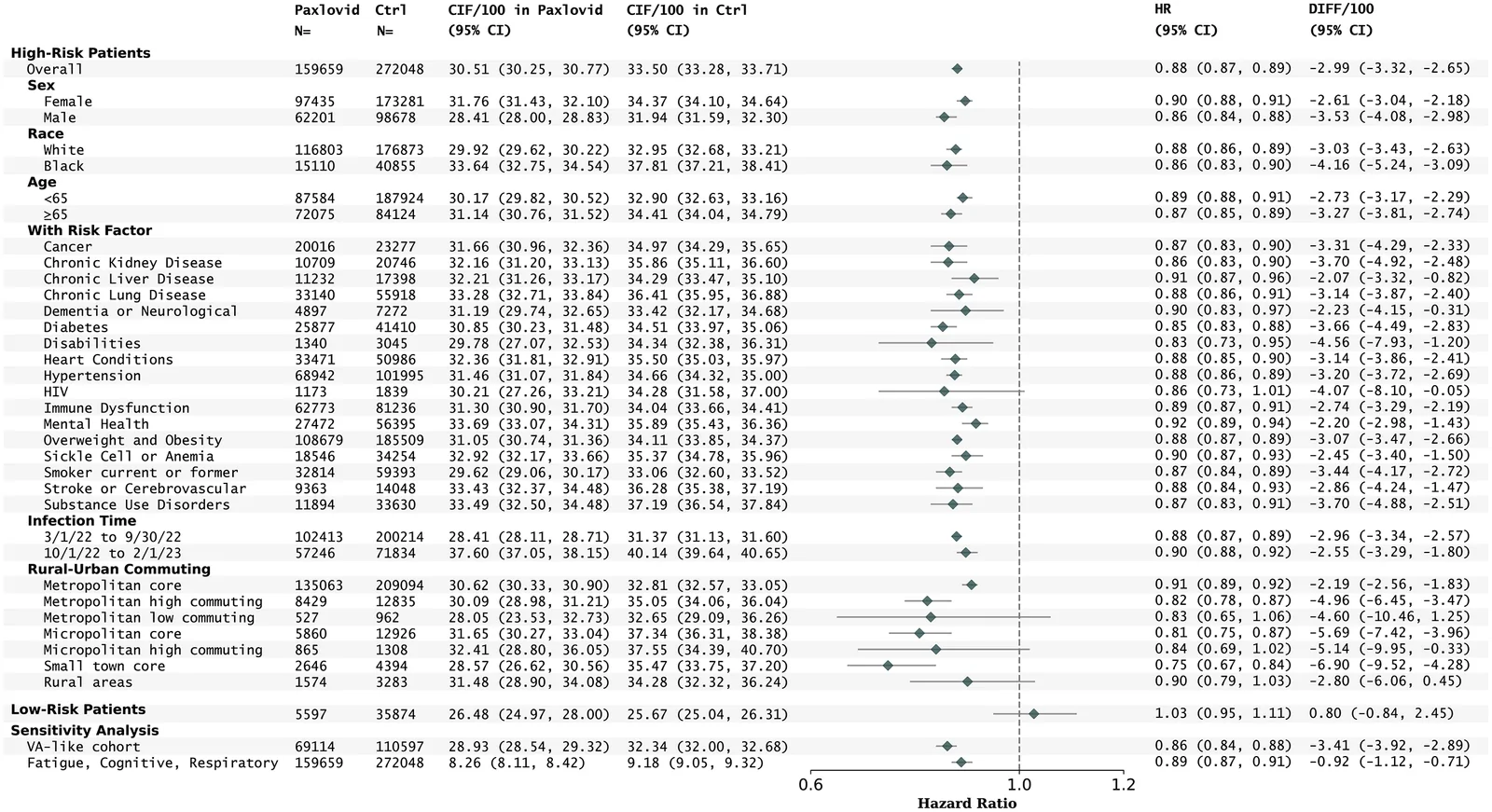
Risks of Long COVID in subgroups of high-risk patients (stratified by sex, race, age, coexisting conditions, infection time, and rural-urban areas), low risk patients, and sensitivity analyses by using different cohort and Long COVID definitions. The absolute risk, risk ratio, and risk difference were captured by the cumulative incidence (CIF), hazard ratio (HR), and the difference of cumulative incidence per 100 persons (DIFF/100), estimated at 180 days after the infection index date, respectively. Shaded areas represent pointwise 95% Confidence Intervals (95% CI).

However, in low-risk patients who didn’t have documented risk factors for severe COVID-19 illness or who were younger than 50, the association between Paxlovid treatment and incident Long COVID was inconclusive (HR, 1.03; 95% CI, 0.95-1.11).

## Discussion

In this clinical trial emulation of 497,499 individuals with diagnosed SARS-CoV-2 infection between March 1, 2022, and February 28, 2023, we found that Paxlovid treatment within 5 days of diagnosis was associated with a reduced risk of developing a range of post-acute conditions in various organ systems, among patients at high-risk for severe COVID-19 illness. Individuals treated with Paxlovid were also significantly less likely to be hospitalized or to die from any cause in the post-acute phase. These results suggest that Paxlovid may significantly reduce the risk of Long COVID and other downstream consequences of acute COVID-19 infection for high-risk non-hospitalized adults. Current guidelines recommend the use of Paxlovid for treating acute COVID-19 [9]; our findings suggest that its potential to reduce the risk of Long COVID conditions among high-risk patients should also be considered.

We did not, however, observe a reduction in Long COVID risk among patients who did not have risk factors for severe COVID-19. This finding is consistent with a recent randomized clinical trial that failed to show a significant benefit of Paxlovid in treatment of acute COVID-19 in low-risk populations [23]. Taken together, this suggests limited utility of Paxlovid in low-risk patients, both for preventing short-term and long-term consequences of infection [9]. In subgroup analyses, the magnitude of benefit appeared smaller and was not statistically significant among individuals at lower baseline risk. This likely reflects the lower baseline risk of severe post-acute outcomes in these populations, which reduces the absolute benefit achievable through antiviral therapy and limits statistical power to detect differences.

This study has several strengths. First, it is the largest observational cohort study examining the impact of Paxlovid on Long COVID conditions [13]. The study’s diverse demographic composition allows for more generalizable conclusions and allows for a more granular investigation of the effect of Paxlovid in various patient subgroups. Our study suggests that, compared to the veteran population [13], the beneficial effects of acute Paxlovid treatment on Long COVID can be extended to female patients, Black patients, and patients who live in metropolitan areas. Importantly there is no evidence of benefit of Paxlovid in preventing Long COVID for pregnant females and low-risk patients. Furthermore, though the beneficial effects span across different subgroups, greater risk reductions were observed in males compared to females, patients aged 65 years and older compared to those under 65, and rural patients compared to micropolitan and metropolitan patients. Second, this study included a broad set of Long COVID outcomes including post-acute incident conditions involving multiple organ systems, post-acute hospitalization, and death. Our results demonstrate that Paxlovid treatment is associated with reduced risk in most outcomes, especially in post-acute hospitalization, death, pulmonary embolism, dementia, and thromboembolism. One exception is the less severe outcome of hair loss which had no significant association with Paxlovid use. The benefits of Paxlovid treatment on Long COVID may be due to reduced acute severity of SARS-CoV-2 infection but differential effects might arise from distinct biologic mechanisms of different Long COVID subtypes [2,6].

Our study also has limitations. First, this was an observational analysis, and patients were not randomized to treatment or control groups. However, the baseline covariates and negative outcome control analyses suggest relatively good balance between the groups; however, residual confounding may still exist. Second, we considered only incident conditions in the post-acute period and did not examine worsening or prolonging of preexisting conditions after the COVID-19 infection. Third, the study used structured information in EHR, which may be incomplete and does not capture patients who do not seek or access care and, on some variables, defined based on ICD codes and EHR data may introduce misclassification bias. Fourth, we estimated intention-to-treatment effects without modeling medication adherence during the acute phase; we could not determine adherence to Paxlovid treatment after prescription.

In conclusion, we conducted the largest observational study to date investigating the effectiveness of Paxlovid treatment within 5 days of SARS-CoV-2 infection in preventing Long COVID in a diverse patient population. Our study suggests that, among high-risk, nonhospitalized patients with COVID-19, treatment with Paxlovid during the acute illness was associated with reduced risk of Long COVID, post-acute hospitalization, and death. These benefits do not appear among low-risk patients.

## Supporting information

S1 Appendix Supplementary Appendix. (DOCX)
