# Supplementary material for: Real-world effectiveness of Paxlovid in protecting Long COVID for outpatient adult patients: A target trial emulation study from the RECOVER Initiative
Source: PLOS Glob Public Health. 2026 Oct 28;6(10):e0005771. doi: 10.1371/journal.pgph.0005771 (PMC13716174; doi:10.1371/journal.pgph.0005771)
Supplement: Supplementary file 1 [file pgph.0005771.s001.docx]

Supplementary Appendix

# **Acknowledgments**

This research was funded by the National Institutes of Health (NIH) Agreement OTA. OT2HL161847 (contract number EHR-01-21) as part of the Researching COVID to Enhance Recovery (RECOVER) research program. The PCORnet Study reported in this work was conducted using PCORnet, the National Patient-Centered Clinical Research Network. PCORnet has been developed with funding from the Patient-Centered Outcomes Research Institute (PCORI). The content is solely the responsibility of the authors and does not necessarily represent the official views of the RECOVER Program, the NIH or other funders. Authorship has been determined according to ICMJE recommendations. We would like to thank the National Community Engagement Group (NCEG), all patient, caregiver and community Representatives, and all the participants enrolled in the RECOVER Initiative. To view the full list of core contributors and data contributors, please refer to the RECOVER-EHR Consortium Members list in the appendix below.

# **RECOVER-EHR Consortium Members**

PCORnet Core Contributors

Louisiana Public Health Institute: *Tom Carton, PI,* Anna Legrand, Elizabeth Nauman

Weill Cornell Medicine: *Rainu Kaushal, PI,* Sajjad Abedian, Dominique Brown, Christopher Cameron, Thomas Campion, Andrea Cohen, Marietou Dione, Rosie Ferris, Wilson Jacobs, Michael Koropsak, Alex LaMar*,* Colby V. Lewis*,* Dmitry Morozyuk*,* Peter Morrisey, Duncan Orlander, Jyotishman Pathak, Mahfuza Sabiha, Edward J. Schenck, Stephenson Strobel, Zoe Verzani, Fei Wang, Mark Weiner, Zhenxing Xu, Chengxi Zang, Yongkang Zhang

PCORnet Data Contributors

Albert Einstein College of Medicine *Parsa Mirhaji, PI |* Columbia University *Soumitra Sengupta, PI,* Karthik Natarajan *|* Duke University Health System *William Schuyler Jones, PI,* Curtis Kieler *|* Emory University *Rishi Kamaleswaran, PI,* Nita Deshpande *|* Icahn School of Medicine at Mount Sinai *Carol R. Horowitz, PI |* Intermountain Healthcare Channing Hansen *|* Intermountain Medical Center Heart Institute *Benjamin D. Horne, PI |* Medical College of Wisconsin *Reza Shaker, PI, Bradley Taylor, PI,* Alexander Stoddard *|* Medical University of South Carolina *Leslie Lenert, PI |* Nicklaus Children's Hospital *Sandy Gonzalez, PI,* Maurice Duque *|* New York University Langone Health *Saul Blecker, PI,* Claudia Pulgarin *|* OCHIN, Inc. *Marion R. Sills, PI |* Ochsner Health System *Daniel Fort, PI |* Penn State U College of Medicine *Cynthia H. Chuang, PI,* Wenke Hwang *|* Temple University Aaron Mishkin *|* The Ohio State University *Soledad A. Fernandez, PI,* Neena Anne Thomas *|* University Medical Center New Orleans *Yuriy Bisyuk, PI |* University of California San Francisco *Susan Kim, PI,* Mark Pletcher *|* University of Florida *Mei Liu, PI,* Jiang Bian *|* University of Iowa *Elizabeth A. Chrischilles, PI,* Boyd Knosp *|* University of Miami *Nick Tsinoremas, PI |* University of Michigan *David A. Williams, PI |* University of Missouri School of Medicine *Abu Saleh Mohammad Mosa, PI,* Xing Song *|* University of Nebraska Medical Center A. Jerrod Anzalone *|* University of Pittsburgh *Jonathan Arnold, PI, Michael J. Becich, PI,* Nickie Cappella, Richard Morgan *|* University of South Florida *Asa Oxner, PI,* Athanasios Tsalatsanis *|* University of Texas Southwestern Medical Center *Lindsay Cowell, PI,* Phillip Reeder *|* University of Utah *Mollie R. Cummins, PI,* Ram Gouripeddi *|* Vanderbilt University Medical Center *Yacob Tedla, PI,* Wei-Qi Wei *|* Weill Cornell Medicine *Rainu Kaushal, PI,* Thomas Campion

# **The PCORNet RECOVER Databases**

The PCORnet RECOVER infrastructure leveraged PCORnet to develop a single, unified EHR/RWD repository to study PASC across ~28.25 million (18.75 million adult - 9.5 million pediatric) patients from 40 adult and pediatric health systems nationwide who continue to refresh their data at least quarterly. The source data includes patients tested for COVID-19 (regardless of result), those diagnosed with COVID-19, those who received COVID-19 vaccine and therapeutics (e.g., Remdesivir and Paxlovid), and/or those who have received a respiratory diagnosis since 2019. The enclave contains structured EHR data consisting of inpatient and ambulatory encounters, laboratory results, vital signs, medications, diagnoses, procedures, birthdates, gender, and race information. The EHR data is linked to geocoded data to the level of the census tract, block group, and/or 9-digit zip code to allow linkage to exposome information to assess the influence of SDoH and environmental exposures on COVID-19 outcomes. In addition, our data enclave includes clinical notes for NLP, vaccine registries, and death registries.

# **Long COVID definition**

The PCORnet’s Long COVID definition for adults is rules-based by leveraging ICD-10 codes covering 24 clinical symptoms, including:

1. sleep wake disorders,
2. cognitive problems,
3. headache,
4. encephalopathy,
5. dementia,
6. hair loss,
7. pulmonary fibrosis/edema/inflammation,
8. acute pharyngitis,
9. dyspnea (or shortness of breath),
10. dysrhythmia,
11. chest pain,
12. thromboembolism,
13. pulmonary embolism,
14. anemia,
15. edema,
16. diabetes mellitus,
17. malnutrition,
18. abdominal pain,
19. constipation,
20. joint pain,
21. fever,
22. malaise and fatigue
23. smell and taste,
24. ICD-10 codes U09.9/B94.8 (representing Post COVID-19 condition, unspecified),

based on prior works.^1–3^ The incident post-acute sequelae of SARS-CoV-2 (PASC, or Long COVID) symptoms from the list above were observed during the post-acute phase (30 to 180 days after the index date), while absent during the baseline period (7 days to 3 years preceding the index date). Individuals were classified as having incident PASC/Long COVID when he/she exhibited at least one incident symptom during the post-acute phase. See the detailed ICD-10 list for each condition in the supplementary materials.

Our secondary definition of Long COVID used in sensitivity analysis focused on fatigue, cognitive, and respiratory symptom clusters, ^4^ also leveraging ICD-10 codes (eTable 2 in Ref^4^ Supplemental Online Content).

# **Fig A. Covariate balance plot before versus after balancing from the Paxlovid-exposed group versus the non-exposed group from the adult SARS-CoV-2 infected patients who were at risk of developing severe COVID-19 illness, during the period from March 1, 2022, to February 28, 2023.** No significant difference was assumed if the standardized mean difference (SMD) was within ±0.1.

# **Fig B. Covariate balance plot before versus after balancing from the Paxlovid-exposed group versus the non-exposed group from the adult SARS-CoV-2 infected patients who were** $\boldsymbol{\leq}$ **50 years old and had no documented risk factors of developing severe COVID-19 illness, during the period from March 1, 2022, to February 28, 2023.** No significant difference was assumed if the standardized mean difference (SMD) was within ±0.1.

# **Fig C. Negative outcome control 1. The cumulative incidence of incident benign neoplasms in the post-acute phase among the Paxlovid-exposed group versus the non-exposed group from the adult SARS-CoV-2 infected patients who were at risk of developing severe COVID-19 illness, during the period from March 1, 2022, to February 28, 2023.** The absolute difference between cumulative incidences and the hazard ratio were computed on day 180 after the infection.

# **Fig D. Negative outcome control 2. The cumulative incidence of incident skin cancer in the post-acute phase among the Paxlovid-exposed group versus the non-exposed group from the adult SARS-CoV-2 infected patients who were at risk of developing severe COVID-19 illness, during the period from March 1, 2022, to February 28, 2023.** The absolute difference between cumulative incidences and the hazard ratio were computed on day 180 after the infection.

# **Fig E. Absolute Risk, Risk Ratio, Risk Difference, and associated P-Values of Long COVID in the Paxlovid group versus the Non-treated group at 180 days after the infection.** Outcomes were ascertained 30 days after the first documented SARS-CoV-2 infection evidence until the end of the follow-up. The absolute risk, risk ratio, and risk difference were captured by the cumulative incidence (CIF), hazard ratio (HR), and the difference of cumulative incidence per 100 persons (DIFF/100), estimated at 180 days after the infection index date, respectively. Shaded areas represent pointwise 95% Confidence Intervals (95% CI). The hazard ratio and its P-value were calculated by the Cox proportional hazard model and the Wald Chi-Square test. The difference in cumulative incidence was tested at 180 days using a transformed survival function and a chi-square test described in ^5^.

# **Fig F. Absolute Risk, Risk Ratio, and Risk Difference of documented Post-acute Death in different subgroups stratified by sex, race, age, coexisting conditions, infection time, and rural-urban areas, and in VA-like cohort.** The absolute risk, risk ratio, and risk difference were captured by the cumulative incidence (CIF), hazard ratio (HR), and the difference of cumulative incidence per 100 persons (DIFF/100), estimated at 180 days after the infection index date, respectively. Shaded areas represent pointwise 95% Confidence Intervals (95% CI). in patients without documented risk factors or risk characteristics.

# **Fig G. Absolute Risk, Risk Ratio, and Risk Difference of Post-acute Hospitalization in different subgroups stratified by sex, race, age, coexisting conditions, infection time, and rural-urban areas, in patients without documented risk factors or risk characteristics, and in VA-like cohort.** The absolute risk, risk ratio, and risk difference were captured by the cumulative incidence (CIF), hazard ratio (HR), and the difference of cumulative incidence per 100 persons (DIFF/100), estimated at 180 days after the infection index date, respectively. Shaded areas represent pointwise 95% Confidence Intervals (95% CI).

# **Fig H. Absolute Risk, Risk Ratio, and Risk Difference of Post-acute Fatigue, Cognitive, and Respiratory condition cluster in different subgroups stratified by sex, race, age, coexisting conditions, infection time, and rural-urban areas, in patients without documented risk factors or risk characteristics, and in VA-like cohort.** The absolute risk, risk ratio, and risk difference were captured by the cumulative incidence (CIF), hazard ratio (HR), and the difference of cumulative incidence per 100 persons (DIFF/100), estimated at 180 days after the infection index date, respectively. Shaded areas represent pointwise 95% Confidence Intervals (95% CI).

# **Table A. Protocol of the target trial and its emulation**

| **Table A.** Protocol specifications of a target trial and its emulation to evaluate the effectiveness of the acute prescription of Paxlovid on the Long COVID,^a^ using observational data from PCORNet Recover Databases, during the period from March 1, 2022, to February 28, 2023 | | |
| --- | --- | --- |
| **Protocol**  **component** | **Target trial** | **Emulation** |
| Eligibility criteria | Inclusion criteria:   - Aged ≥18 years at the first confirmed SARS-CoV-2 infection between March 1, 2022, and February 28, 2023 - Interactions with the healthcare system in the past 3 years to 7 days before the first infection - Either at increased risk for developing severe COVID-19 illness. At-risk patients were identified by age ≥50 years or having at least 1 underlying medical condition^b^ associated with an increased risk of developing severe illness from COVID-19   Exclusion criteria:   - History of Long COVID or sequelae of other specified infectious and parasitic diseases - Hospitalized at the infection or need for hospitalization for the medical treatment of COVID-19 - Known medical history of severe liver disease - Receiving dialysis or having severe renal impairment - Has received or is expected to receive other COVID-19 treatments including antiviral, antibody, or convalescent plasma treatments - Has received or is expected to receive contraindicated drugs for Paxlovid^c^ | Same as for the target trial, except:   - We identified the initial SARS-CoV-2 infected patients if they had any a) positive SARS-CoV-2 polymerase-chain-reaction (PCR) or antigen laboratory tests; b) the International Classification of Diseases, Tenth Revision, Clinical Modification (ICD-10-CM) diagnosis code U07.1 representing COVIID-19 diagnosis; or c) Paxlovid (nirmatrelvir/ritonavir) or Remdesivir prescriptions. The date of the first documented infection evidence was considered as the COVID-19 index date. - Having received or being expected to receive other COVID-19 treatments or contraindicated drugs was ascertained during -14 days to 14 days around the COVID-19 index date. - Having hospitalized for the infection or need for hospitalization for medical treatment, including ventilation and ICU care, of COVID-19 as well for were ascertained during -1 day to 14 days around the COVID-19 index date. |
| Treatment strategies | - Exposed arm: receive Paxlovid within 5 days of the confirmed SARS-CoV-2 infection, or - Control arm: receive placebo within 5 days of the confirmed SARS-CoV-2 infection | Same as for the target trial, except:   - The control arm consisted of patients without documented Paxlovid treatment, and according to the eligibility criteria, not having other COVID-19 treatments during -1 day to 14 days around the COVID-19 index date either.   The index date for treated group was defined as the date of the first Paxlovid prescription and the index date for the control group was defined as the date of the first documented evidence of SARS-CoV-2 infection |
| Treatment assignment | Individuals are randomly assigned to a strategy at the baseline and will be aware of the assigned treatment strategy. | We classified individuals according to the treatment strategies that their data were compatible with at baseline and attempted to emulate randomization by adjusting for baseline confounders |
| Outcomes | - Post-acute death from any cause from day 31 to day 180 - (Symptomatic) post-acute sequelae of SARS-CoV-2 infection (PASC) from day 31 to day 180 - Post-acute hospitalization from any cause from day 31 to day 180 | Same as for the target trial. |
| Follow-up | For each person, follow-up starts on the day 31 after the SARS-CoV-2 infection (baseline) and ends on the day of the post-acute outcome of interest, death, 180 days after baseline, loss of follow-up, or the end of the study period (August 1, 2023), whichever happens first. | Same as for the target trial. |
| Causal contrasts | Intention-to-treat effect. | Observational analog of intention-to-treat effect |
| Statistical analysis | - Cumulative incidence (risk) curves and estimates of risk, risk differences, risk ratios, and hazard ratios comparing treated versus non-treated groups at 180 days after the baseline. - Subgroup analyses by baseline age, race, and underlying risk conditions. | Same intention-to-treat analyses with:   - Adjusted cumulative incidence (risk) curves and estimates of risk, risk differences, risk ratios, and adjusted hazard ratios comparing treated versus non-treated groups at 180 days after the baseline. Baseline covariates and stabilized inverse probability of treatment weights were used for the adjusted analyses. The cumulative incidence functions were estimated by the Aalen-Johansen method, and the hazard ratios were estimated by the Cox proportional hazard model. The death event, both in acute and post-acute phases, was modeled as a competing event that precludes other outcomes of interest from occurring. - Subgroup analyses by baseline age, race, and underlying risk conditions. - Sensitivity analyses by using different PASC/Long COVID definitions (fatigue cognitive and respiratory symptom clusters), US Veterans study-like cohort (87.8% male patients aged ≥60 years old or having any risk factors), and potential different variants of concerns (approximated by infection time before versus after October 1, 2022, in the study period) - Negative outcome control analysis to test potential residual confoundings |

a. The hypothetical target trial tried to mimic the trial NCT04960202 <https://classic.clinicaltrials.gov/ct2/show/NCT04960202> with a focus on the Long COVID outcome. protocols were

b. See the full list of risk factors at <https://www.cdc.gov/coronavirus/2019-ncov/need-extra-precautions/people-with-medical-conditions.html> and <https://www.paxlovidinformation.com/who-can-take>

c. See contraindicated drugs at <https://www.paxlovidinformation.com/who-can-take>.

# **Table B. Study covariates**

| **Table B.** Covariates of the target trial emulation to evaluate the effectiveness of the acute prescription of Paxlovid on the PASC conditions using observational data from PCORNet Recover Databases, during the period from March 1, 2022, to February 28, 2023, | | | |
| --- | --- | --- | --- |
| **Variable** | **Functional form** | **Values** | **Details** |
| Sex | Categories | Male/Female/Other or missing | Based on records in the Demographic Domain |
| Age | Categories | 18-24, 25-34, 35-49, 50-64, 65-74, 75-84, ≥85 were used in primary analysis. 18-24, 25-29, 30-34, 35-39, 40-44, 45-50 were used for patients ≤ 50 years old and had no documented risk factors. | Based on records in the Demographic Domain. The age at the index date was captured. |
| Race/Ethnicity | Categories | Asian Non-Hispanic,  Black or African American Non-Hispanic,  Hispanic or Latino Any Race,  White Non-Hispanic,  Other Non-Hispanic,  Unknown race/ethnicity | Based on records in the Demographic Domain. |
| Area Deprivation Index (ADI) | Categories | 1-9, 10-19, 20-29, 30-39, 40-49, 50-59, 60-69, 70-79, 80-89, 90-100, Missing | Based on the Federal Information Processing Series (FIPS) codes in the Geographic code domain from 2020 and the most recent address and ZIP code records in the Longitudinal Address history Domain. |
| Infection time periods | Categories | 03/2022-06/2022, 07/2022-10/2022, 11/2022-02/2023 | Based on the index date. The SARS-CoV-2 infection evidence was based on a) positive SARS-CoV-2 polymerase-chain-reaction (PCR) or antigen laboratory tests in the Lab Result domain; b) the International Classification of Diseases, Tenth Revision, Clinical Modification (ICD-10-CM) diagnosis code U07.1 representing COVIID-19 diagnosis in the Diagnosis domain; or c) Paxlovid (nirmatrelvir/ritonavir) or Remdesivir prescriptions in the Prescribing domain, Dispensing domain, and the Administered Medications domain. The date of the first documented infection evidence was considered as the COVID-19 index date. |
| History hospital utilization | Categories | 0 inpatient visits, 1-2 inpatient visits, 3-4 inpatient visits, ≥5 inpatient visits, 0 outpatient visits, 1-2 outpatient visits, 3-4 outpatient visits, ≥5 outpatient visits, 0 emergency visits, 1-2 emergency visits, 3-4 emergency visits, ≥5 emergency visits | Based on the Encounter domain and history of encounter types in the past 3 years before the index date. |
| Body mass index (kg/m^2^) | Categories | <18.5 underweight,  18.5 to <25 normal weight, 25 to <30 overweight, ≥30 obese, and missing, | Based on the most recent records in the Vital domain. |
| Smoking status | Categories | Never, current, former, and missing | Based on the most recent records in the Vital domain. |
| COVID-19 vaccine status | Categories | Fully vaccinated, partially vaccinated, no evidence | Based on the Procedures domain and Immunization domain. |
| Cancer | Indicator | Yes/No | Based on the Diagnosis domain in the past 3 years before the index |
| Chronic kidney disease | Indicator | Yes/No | Based on the Diagnosis domain in the past 3 years before the index |
| Chronic liver disease | Indicator | Yes/No | Based on the Diagnosis domain in the past 3 years before the index |
| Chronic lung disease | Indicator | Yes/No | Based on the Diagnosis domain in the past 3 years before the index |
| Cystic fibrosis | Indicator | Yes/No | Based on the Diagnosis domain in the past 3 years before the index |
| Dementia or other neurological conditions | Indicator | Yes/No | Based on the Diagnosis domain in the past 3 years before the index |
| Diabetes | Indicator | Yes/No | Based on the Diagnosis domain in the past 3 years before the index |
| Disabilities | Indicator | Yes/No | Based on the Diagnosis domain in the past 3 years before the index |
| Heart conditions | Indicator | Yes/No | Based on the Diagnosis domain in the past 3 years before the index |
| Hypertension | Indicator | Yes/No | Based on the Diagnosis domain in the past 3 years before the index |
| Pregnant | Indicator | Yes/No | Based on the Diagnosis domain, Procedures domain, and Encounter domain, the index date was required in the gestational period before the delivery event. |
| HIV infection | Indicator | Yes/No | Based on the Diagnosis domain in the past 3 years before the index |
| Immune dysfunction | Indicator | Yes/No | Based on the Diagnosis domain, Prescribing domain, Dispensing domain, and Administered Medications domain in the past 3 years before the index |
| Mental health conditions | Indicator | Yes/No | Based on the Diagnosis domain in the past 3 years before the index |
| Overweight and obesity | Indicator | Yes/No | Based on the Diagnosis domain the past 3 years before the index and the most recent record in the Vital domain in |
| Sickle cell disease or thalassemia or anemia | Indicator | Yes/No | Based on the Diagnosis domain in the past 3 years before the index |
| Smoking current or former | Indicator | Yes/No | Based on the most recent record in the Vital domain |
| Stroke or cerebrovascular disease | Indicator | Yes/No | Based on the Diagnosis domain in the past 3 years before the index |
| Substance use disorders | Indicator | Yes/No | Based on the Diagnosis domain in the past 3 years before the index |
| Tuberculosis | Indicator | Yes/No | Based on the Diagnosis domain in the past 3 years before the index |
| Severe liver disease | Indicator | Yes/No | Based on the Diagnosis domain in the past 3 years before the index |
| Severe kidney disease or on dialysis | Indicator | Yes/No | Based on the Diagnosis domain and Procedures domain in the past 3 years before the index |
| Coagulopathy | Indicator | Yes/No | Based on the Diagnosis domain in the past 3 years before the index |
| Peripheral vascular disorders | Indicator | Yes/No | Based on the Diagnosis domain in the past 3 years before the index |
| Seizure or Epilepsy | Indicator | Yes/No | Based on the Diagnosis domain in the past 3 years before the index |
| Weight Loss | Indicator | Yes/No | Based on the Diagnosis domain in the past 3 years before the index |
| Obstructive sleep apnea | Indicator | Yes/No | Based on the Diagnosis domain in the past 3 years before the index |
| Epstein-Barr and Infectious Mononucleosis | Indicator | Yes/No | Based on the Diagnosis domain and Lab results domain in the past 3 years before the index |
| Herpes Zoster | Indicator | Yes/No | Based on the Diagnosis domain in the past 3 years before the index |
|  |  |  |  |

a. See the specification of PCORNet common data model and domains at <https://pcornet.org/data/>

# **Table C. Sensitivity analysis of comparing primary results with bootstrapping results and percentile-based 95% confidence intervals for the risks and risk reductions.**

|  | Primary Results | | | | Bootstrapping Results | | | |
| --- | --- | --- | --- | --- | --- | --- | --- | --- |
|  | CIF/100 in Paxlovid (95% CI) | CIF/100 in Ctrl (95% CI) | DIFF/100 (95% CI) | Chi squared test of difference | CIF/100 in Paxlovid (95% CI) | CIF/100 in Ctrl (95% CI) | DIFF/100 (95% CI) | Chi squared test of difference |
| Long COVID | 30.51 (30.25, 30.77) | 33.50 (33.28, 33.71) | -2.99 (-3.32, -2.65) | 8.80E-140 | 30.52 (30.25, 30.79) | 33.50 (33.27, 33.74) | -2.98 (-3.33, -2.60) | 3.70E-123 |
| Death | 0.28 (0.25, 0.31) | 0.52 (0.48, 0.55) | -0.23 (-0.28, -0.19) | 8.40E-22 | 0.28 (0.25, 0.31) | 0.52 (0.48, 0.55) | -0.23 (-0.28, -0.18) | 9.70E-20 |
| Hospitalization | 6.06 (5.93, 6.20) | 8.44 (8.31, 8.56) | -2.37 (-2.56, -2.19) | 1.60E-148 | 6.07 (5.92, 6.23) | 8.44 (8.29, 8.57) | -2.37 (-2.57, -2.16) | 1.70E-120 |
| Dementia | 0.26 (0.23, 0.29) | 0.34 (0.32, 0.37) | -0.09 (-0.12, -0.05) | 4.00E-05 | 0.26 (0.23, 0.29) | 0.34 (0.31, 0.37) | -0.09 (-0.12, -0.05) | 7.10E-05 |
| Encephalopathy | 0.74 (0.69, 0.79) | 0.89 (0.85, 0.94) | -0.15 (-0.22, -0.08) | 9.20E-06 | 0.74 (0.69, 0.79) | 0.89 (0.84, 0.93) | -0.15 (-0.22, -0.08) | 3.80E-05 |
| Headache | 2.64 (2.55, 2.75) | 2.91 (2.82, 3.00) | -0.27 (-0.40, -0.13) | 6.00E-05 | 2.65 (2.53, 2.76) | 2.92 (2.83, 3.00) | -0.27 (-0.41, -0.13) | 1.60E-04 |
| Cognitive problems | 2.74 (2.64, 2.84) | 2.96 (2.88, 3.04) | -0.22 (-0.35, -0.09) | 6.90E-04 | 2.74 (2.63, 2.84) | 2.96 (2.87, 3.05) | -0.22 (-0.35, -0.08) | 0.002 |
| Sleep disorders | 3.45 (3.33, 3.57) | 3.63 (3.53, 3.73) | -0.18 (-0.33, -0.03) | 0.018 | 3.45 (3.32, 3.58) | 3.63 (3.53, 3.74) | -0.18 (-0.34, -0.02) | 0.023 |
| Hair loss | 0.75 (0.70, 0.80) | 0.72 (0.68, 0.76) | 0.03 (-0.03, 0.10) | 0.326 | 0.75 (0.70, 0.80) | 0.72 (0.68, 0.77) | 0.03 (-0.04, 0.09) | 0.379 |
| Shortness of breath | 8.86 (8.66, 9.07) | 9.89 (9.72, 10.07) | -1.03 (-1.30, -0.76) | 5.20E-16 | 8.86 (8.65, 9.09) | 9.90 (9.71, 10.08) | -1.04 (-1.32, -0.75) | 5.00E-14 |
| Acute pharyngitis | 3.96 (3.84, 4.08) | 4.42 (4.32, 4.53) | -0.46 (-0.63, -0.30) | 7.40E-09 | 3.95 (3.82, 4.08) | 4.42 (4.32, 4.53) | -0.47 (-0.64, -0.30) | 4.60E-08 |
| Pulmonary fibrosis | 1.15 (1.09, 1.22) | 1.27 (1.22, 1.32) | -0.12 (-0.20, -0.03) | 0.006 | 1.15 (1.08, 1.22) | 1.27 (1.21, 1.32) | -0.12 (-0.20, -0.03) | 0.008 |
| Pulmonary embolism | 0.19 (0.17, 0.22) | 0.29 (0.26, 0.31) | -0.09 (-0.13, -0.06) | 4.90E-07 | 0.19 (0.17, 0.22) | 0.29 (0.26, 0.31) | -0.09 (-0.13, -0.06) | 2.90E-06 |
| Thromboembolism | 0.34 (0.31, 0.37) | 0.44 (0.41, 0.47) | -0.10 (-0.14, -0.05) | 2.90E-05 | 0.34 (0.30, 0.37) | 0.44 (0.41, 0.47) | -0.10 (-0.15, -0.05) | 6.70E-05 |
| Chest pain | 3.26 (3.15, 3.37) | 3.74 (3.64, 3.84) | -0.48 (-0.62, -0.33) | 6.00E-11 | 3.26 (3.14, 3.39) | 3.74 (3.64, 3.84) | -0.48 (-0.63, -0.31) | 1.60E-09 |
| Dysrhythmia | 3.89 (3.76, 4.01) | 4.24 (4.14, 4.35) | -0.36 (-0.52, -0.20) | 6.80E-06 | 3.89 (3.76, 4.02) | 4.24 (4.14, 4.36) | -0.36 (-0.52, -0.20) | 1.70E-05 |
| Anemia | 2.29 (2.20, 2.38) | 2.78 (2.70, 2.86) | -0.49 (-0.61, -0.37) | 8.70E-16 | 2.29 (2.19, 2.39) | 2.78 (2.70, 2.87) | -0.49 (-0.63, -0.36) | 4.90E-13 |
| Malnutrition | 0.30 (0.27, 0.34) | 0.47 (0.44, 0.50) | -0.16 (-0.21, -0.12) | 4.60E-12 | 0.30 (0.27, 0.34) | 0.47 (0.44, 0.51) | -0.17 (-0.22, -0.12) | 4.10E-11 |
| Diabetes | 1.24 (1.18, 1.32) | 1.42 (1.36, 1.48) | -0.18 (-0.27, -0.08) | 1.50E-04 | 1.25 (1.17, 1.32) | 1.42 (1.36, 1.48) | -0.17 (-0.27, -0.07) | 5.00E-04 |
| Edema | 5.30 (5.15, 5.45) | 6.00 (5.87, 6.13) | -0.69 (-0.89, -0.49) | 8.60E-13 | 5.31 (5.15, 5.47) | 5.99 (5.85, 6.12) | -0.68 (-0.90, -0.48) | 1.30E-10 |
| Constipation | 2.26 (2.17, 2.35) | 2.64 (2.56, 2.72) | -0.38 (-0.50, -0.26) | 3.10E-10 | 2.26 (2.16, 2.35) | 2.64 (2.56, 2.73) | -0.39 (-0.52, -0.26) | 2.20E-09 |
| Abdominal pain | 6.50 (6.33, 6.67) | 7.42 (7.28, 7.58) | -0.92 (-1.15, -0.70) | 2.80E-17 | 6.51 (6.31, 6.70) | 7.42 (7.26, 7.58) | -0.92 (-1.16, -0.68) | 1.80E-14 |
| Joint pain | 8.41 (8.19, 8.62) | 9.68 (9.49, 9.86) | -1.27 (-1.55, -0.99) | 1.60E-21 | 8.41 (8.17, 8.64) | 9.67 (9.48, 9.87) | -1.26 (-1.56, -0.96) | 1.40E-18 |
| Smell and taste | 0.11 (0.10, 0.13) | 0.14 (0.13, 0.16) | -0.03 (-0.06, -0.00) | 0.03 | 0.11 (0.10, 0.13) | 0.14 (0.13, 0.16) | -0.03 (-0.06, -0.00) | 0.031 |
| Fever | 1.01 (0.95, 1.07) | 1.23 (1.17, 1.28) | -0.22 (-0.29, -0.14) | 1.20E-07 | 1.01 (0.95, 1.08) | 1.23 (1.17, 1.28) | -0.22 (-0.30, -0.12) | 1.80E-06 |
| Malaise and fatigue | 3.69 (3.57, 3.81) | 4.28 (4.18, 4.39) | -0.59 (-0.75, -0.43) | 1.10E-13 | 3.70 (3.57, 3.83) | 4.28 (4.17, 4.40) | -0.58 (-0.76, -0.40) | 1.90E-11 |
| U099/B948 | 0.90 (0.85, 0.95) | 0.92 (0.88, 0.97) | -0.03 (-0.09, 0.04) | 0.46 | 0.90 (0.84, 0.95) | 0.92 (0.88, 0.97) | -0.03 (-0.10, 0.04) | 0.487 |

1000-iterations of bootstrapping estimates and percentile-based 95% confidence intervals of stabilized inverse probability reweighted cumulative incidence in different exposure groups were estimated.^6^ The test of risk difference was conducted at 180 days.^5^ CIF, cumulative incidence function, or (absolute risk); DIFF, difference of cumulative incidence, or risk reduction; 95% CI, 95% Confidence Intervals.

# **Reference**

1.	Zhang H, Zang C, Xu Z, et al. Data-driven identification of post-acute SARS-CoV-2 infection subphenotypes. Nat Med 2022;1–10.

2.	Zang C, Zhang Y, Xu J, et al. Data-driven analysis to understand long COVID using electronic health records from the RECOVER initiative. Nat Commun 2023;14(1):1948.

3.	Thaweethai T, Jolley SE, Karlson EW, et al. Development of a Definition of Postacute Sequelae of SARS-CoV-2 Infection. JAMA [Internet] 2023 [cited 2023 May 25];Available from: https://doi.org/10.1001/jama.2023.8823

4.	Global Burden of Disease Long COVID Collaborators. Estimated Global Proportions of Individuals With Persistent Fatigue, Cognitive, and Respiratory Symptom Clusters Following Symptomatic COVID-19 in 2020 and 2021. JAMA [Internet] 2022 [cited 2022 Oct 11];Available from: https://doi.org/10.1001/jama.2022.18931

5.	Klein JP, Logan B, Harhoff M, Andersen PK. Analyzing survival curves at a fixed point in time. Statistics in Medicine 2007;26(24):4505–19.

6.	Austin PC. Variance estimation when using inverse probability of treatment weighting (IPTW) with survival analysis. Statistics in Medicine 2016;35(30):5642–55.
