# Supplementary material for: Real-world effectiveness of Paxlovid in protecting Long COVID for outpatient adult patients: A target trial emulation study from the RECOVER Initiative
Source: PLOS Glob Public Health. 2026 Oct 28;6(10):e0005771. doi: 10.1371/journal.pgph.0005771 (PMC13716174; doi:10.1371/journal.pgph.0005771)
Supplement: Supplementary file 2 [file pgph.0005771.s003.docx]

**­­­Response Letter**

We appreciate the reviewers’ thoughtful comments and provide our point-by-point responses below. We have addressed all the comments of editor and reviewers, followed by our response to the editorial requirements.

CC: ibloom@plos.org

PGPH-D-25-03662

Real World Effectiveness of Nirmatrelvir in Protecting Long COVID for Outpatient Adult Patients: A Large Scale Observational Cohort Study from the RECOVER Initiative

PLOS Global Public Health

Dear Dr. Zang,

Thank you for submitting your manuscript to PLOS Global Public Health. After careful consideration, we feel that it has merit but does not fully meet PLOS Global Public Health’s publication criteria as it currently stands. Therefore, we invite you to submit a revised version of the manuscript that addresses the points raised during the review process.

The manuscript has been evaluated by three reviewers, and their comments are available below.

Could you please carefully revise the manuscript to address all comments raised?

Please submit your revised manuscript by Apr 09 2026 11:59PM. If you will need more time than this to complete your revisions, please reply to this message or contact the journal office at globalpubhealth@plos.org. When you're ready to submit your revision, log on to https://www.editorialmanager.com/pgph/ and select the 'Submissions Needing Revision' folder to locate your manuscript file.

Please include the following items when submitting your revised manuscript:

A letter that responds to each point raised by the editor and reviewer(s). You should upload this letter as a separate file labeled 'Response to Reviewers'.

A marked-up copy of your manuscript that highlights changes made to the original version. You should upload this as a separate file labeled 'Revised Manuscript with Track Changes'.

An unmarked version of your revised paper without tracked changes. You should upload this as a separate file labeled 'Manuscript'.

Guidelines for resubmitting your figure files are available below the reviewer comments at the end of this letter.

We look forward to receiving your revised manuscript.

Kind regards,

Ilse Bloom

Staff Editor

PLOS Global Public Health

Journal Requirements:

1. We ask that a manuscript source file is provided at Revision. Please upload your manuscript file as a .doc, .docx, .rtf or .tex.

Done. submitted in docx

2. We do not publish any copyright or trademark symbols that usually accompany proprietary names, eg (R), (C), or TM (e.g. next to drug or reagent names). Please remove all instances of trademark/copyright symbols throughout the text, including ® on page 8.

Done. Removed

3. Please upload a copy of Figure 1,2 & 3 which you refer to in your text on pages 10,11 & 12. Or, if the figure is no longer to be included as part of the submission please remove all reference to it within the text.

Done. Uploaded as separated files

**Reviewer #1:** Thank you for the opportunity to review the article.

General comments

Recommend following the TARGET Checklist of Recommended Items to Address in Reports of Studies Emulating a Target Trial when reporting the study (https://jamanetwork.com/journals/jama/fullarticle/2837724).

Recommend consistent use of terminology (e.g. nirmatrelvir vs Paxlovid).

**Response:** We thank the reviewer for the helpful suggestions. Following this recommendation, we carefully reviewed our manuscript against the TARGET reporting checklist to ensure that key elements of target trial emulation studies are clearly described, including the specification of eligibility criteria, treatment strategies, assignment procedures, follow-up period, outcomes, and causal contrasts.

We have also standardized the terminology used throughout the manuscript. Specifically, we now consistently refer to the intervention as Paxlovid (or nirmatrelvir–ritonavir) at first mention and use Paxlovid thereafter for clarity and consistency.

Introduction:

Add a clear rationale for emulating a target trial using the available data for this study.

Consider citing the randomized trial(s) that informed the design of the target trial, if available.

**Response:** We have revised the Introduction to more clearly explain the rationale for using a target trial emulation framework in this observational study. “Although studies have shown that Paxlovid is effective at reducing the risk of severe COVID-19, including hospitalization or death,5 limited research exists on whether it is effective in preventing Long COVID, and existing studies have found mixed results.6–8 Further, the association of Paxlovid with Long COVID risk is largely lacking in low-risk patients” In particular, because randomized clinical trials evaluating the impact of nirmatrelvir–ritonavir on *Long COVID* are few, observational electronic health record data provide an important opportunity to examine these associations in real-world populations.

Emulating a target trial allows us to explicitly define key design components, such as eligibility criteria, treatment strategies, assignment procedures, follow-up, and outcomes, in a way that reduces common biases in observational analyses. Following the reviewer’s suggestion, we also clarified that the design of our target trial emulation was informed by the randomized trial. We added detailed specifications of protocols of the target trial, target trial NCT04960202 <https://classic.clinicaltrials.gov/ct2/show/NCT04960202> etc in the *Supplementary Appendix Table S1*, and explicitly referred in the *Method- Study Cohort section*.

Methods:

Clarify how treatment assignment was determined.

**Response:** We have clarified the treatment assignment in the *Methods-Exposure section*. In this observational target trial emulation, treatment assignment was determined based on real-world prescription records in the electronic health record system. “The exposure of interest was receipt of Paxlovid (nirmatrelvir–ritonavir) within 5 days after the SARS-CoV-2 infection index event. Patients were assigned to the treatment group if they had a documented prescription for Paxlovid within this time window. The comparison group consisted of patients who did not receive Paxlovid or other COVID-19 treatments during the acute infection period. Because treatment was not randomly assigned in routine clinical care, we emulated randomization by adjusting for a comprehensive set of baseline covariates using stabilized inverse probability of treatment weighting (IPTW) based on propensity scores.”

Report the frequency of missing data for all variables, stratified by treatment strategy where applicable.

**Response:** We thank the reviewer for the helpful suggestions. In the PCORnet RECOVER data infrastructure, most demographic and clinical variables are standardized through the PCORnet Common Data Model, and key variables used in this study had relatively low levels of missingness. In the revised manuscript, we have clarified how missing covariate data were handled in the statistical analysis. Specifically, missing values in baseline covariates were treated as separate categories for categorical variables when appropriate or handled according to standard PCORnet data conventions as shown in *Table 1 and Table S2 in the appendix*. As shown in Table 1, we reported missing data for sex, race/ethnicity, vaccine etc covariates stratified by treatment strategy.

Inconsistency in study period: Line 144 states March 1, 2022 to February 28, 2023, whereas Figure 1 shows March 1, 2022 to February 1, 2023.

**Response:** Thanks! The study period in this study was March 1, 2022 to *February 28*, 2023 (included). We have corrected the text and figure 1 in the revised manuscript to ensure consistency throughout.

Table 1: consider using “control group” instead of “no treated SARS-CoV-2 Infected.”

**Response:** We have revised Table 1 accordingly by replacing “No treated SARS-CoV-2 Infected Patients” with “Control group” and have ensured consistent terminology throughout the manuscript.

Figure 3 presents two infection-time groups, while Table 1 shows three different index times. Consider conducting subgroup analyses based on the three index-time categories defined in Table 1.

**Response:** In Table 1, the three index-time categories were presented to describe the temporal distribution of infections across the study period every quarter or 4 months. For the subgroup analysis shown in Figure 3, we grouped infection time per different waves of potentially different variants of concerns (approximated by infection time before versus after October 1, 2022, in the study period), see figure below:

We have clarified the rationale for the infection-time grouping in the Methods-statistical analysis section in the revised manuscript as well as Table S1. Protocol of the target trial and its emulation in Appendix.

Supplementary tables are not included in the submission. Please upload all supplementary tables.

**Response:** We have now uploaded all Supplementary Tables as part of the revised submission.

**Reviewer #2:** How was the index date defined?

**Response:** We thank the reviewer for this important question. The index date for treated group was defined as the date of the first Paxlovid prescription and the index date for the control group was defined as the date of the first documented evidence of SARS-CoV-2 infection. See Method-exposure section. Considering the exposure of interest was receipt of Paxlovid (nirmatrelvir–ritonavir) within 5 days after the SARS-CoV-2 infection and the outcome of interests were ascertained 180 days after SARSCoV-2 infection (not happen within 5 days after infection). Thus, immortal bias will not happen.

Provide a clear definition of censoring.

**Response:** We have clarified the definition of censoring in the Method-Outcomes section. In this study, patients were censored at the earliest occurrence of the following events: (1) the incident outcome of interest in the post acute phase (at least 30 days after infection), (2) death (when analyzing non-mortality outcomes), (3) 180 days after infection, or (4) the end of the study period. This clarification has been added to the revised manuscript.

Were standardized mean differences (SMDs) used to assess balance before weighting, after weighting, or both?

How about categorical variables?

**Response:** Both. Standardized mean differences (SMDs) were used to evaluate covariate balance both before (Table 1 in main draft, Figure S1-2 in appendix) and after applying stabilized inverse probability of treatment weighting (IPTW) in Figure S1-2 in appendix.

The SMDs were calculated for both continuous and categorical variables, particularly dichotomous variables, following standard practice, references below, in propensity score analyses.

- Austin, Peter C. "Goodness‐of‐fit diagnostics for the propensity score model when estimating treatment effects using covariate adjustment with the propensity score." Pharmacoepidemiology and drug safety 17.12 (2008): 1202-1217.
- <https://support.sas.com/resources/papers/proceedings12/335-2012.pdf>

We acknowledged that there is another method, using multivariate Mahalanobis distance to handle multinomial variables and summarized them in one value. Instead, we report smd for each subcategory under the multivariate variables, as shown in Fig 1, to better compare each subcategory.

Did the Cox survival model account for competing risks?

**Response:** In our analysis, death was treated as a competing event when estimating cumulative incidence of non-mortality outcomes using the Aalen–Johansen estimator. For the hazard ratio estimation, we used a cause-specific Cox proportional hazards model in the reweighted population. In this framework, individuals who experienced the competing event (death) were censored at the time of death when analyzing non-mortality outcomes. We have clarified this description in the Methods section of the revised manuscript.

Was the proportional hazards assumption tested?

**Response:** In the current analysis, formal tests of the proportional hazards assumption were not conducted.

Per the guidance of JAMA Stensrud, M.J. and Hernán, M.A., 2020. Why test for proportional hazards?. *Jama*, *323*(14), pp.1401-1402,

“statistical tests for proportional hazards are *unnecessary*. Because it is expected that the hazard ratio will vary over the follow-up period, tests of proportional hazards yielding high P values are probably underpowered. *Reports of hazard ratios should be supplemented with reports of effect measures directly calculated from absolute risks, such as the survival differences or the restricted mean survival difference,8 at times prespecified in the study protocol*. These measures are arguably more helpful for clinical decision-making and more easily understood by patients.

Thus, we reported the absolute risk, risk ratio, and risk difference were captured by the cumulative incidence (CIF), hazard ratio (HR), and the difference of cumulative incidence per 100 persons (DIFF/100), estimated at 180 days after the infection date, respectively

How was the regularization parameter (λ) selected?

**Response:** The regularization parameter (λ) in the penalized regression model used to estimate propensity scores was selected using cross-validation. Specifically, λ was chosen based on the value that minimized the cross-validated prediction error on the validation set across 10-fold cross validation process.

Was class imbalance taken into account during model fitting?

**Response:** Thanks for your questions. We fitted the propensity score model for stabilized inverse probability of treatment weighting (IPTW) to reweight the treatment and control groups. We didn’t model the class imbalance in this regard because as shown in the table 1, the eligible treated and control groups are 165256 and 307922, which are comparable in sample size.

What variables were in “all baseline covariates”? Were any variables excluded due to collinearity or missing?

**Response:** The baseline covariates included demographic characteristics (e.g., age, sex, and race/ethnicity), comorbidities, and clinical characteristics measured prior to the index date. The full list of covariates and their assessment windows are provided in Table S2. Because a penalized regression model with regularization was used to estimate propensity scores, variables were not excluded due to collinearity. No covariates were removed due to missingness.

For subgroup analyses, were interaction used or stratified estimation?

**Response:** Thanks for your questions. Subgroup analyses were conducted using stratified estimation. Specifically, the analyses were repeated within each predefined subgroup, and hazard ratios were estimated using separate weighted Cox proportional hazards models within each subgroup. We have clarified this description in the Methods section of the revised manuscript.

Provide details regarding the negative outcome control analyses, including the selection and validation of control outcomes.

**Response:** Negative outcome control analyses were conducted to evaluate the potential presence of residual confounding or systematic bias. Negative control outcomes were selected based on the absence of a known causal relationship with nirmatrelvir exposure and were chosen to represent clinical events unlikely to be affected by antiviral treatment. The selection was informed by clinical knowledge and prior literature. We have clarified the rationale for selecting these outcomes and their role in assessing potential bias in the Methods section of the revised manuscript. Detailed results were shown in Fig S3, S4 in the appendix.

How were missing covariate data handled?

**Response:** We thank the reviewer for the helpful suggestions. In the PCORnet RECOVER data infrastructure, most demographic and clinical variables are standardized through the PCORnet Common Data Model, and key variables used in this study had relatively low levels of missingness. In the revised manuscript, we have clarified how missing covariate data were handled in the statistical analysis. Specifically, missing values in baseline covariates were treated as separate categories for categorical variables when appropriate or handled according to standard PCORnet data conventions as shown in *Table 1 and Table S2 in the appendix*. As shown in Table 1, we reported missing data for sex, race/ethnicity, vaccine etc covariates stratified by treatment strategy. This approach allowed all eligible individuals to be included in the propensity score model and subsequent analyses.

More details are needed on the bootstrapping procedure: Was stratified bootstrapping or simple resampling used?

Were propensity scores re-estimated within each bootstrap iteration? Were IPTW weights recalculated in each iteration?

**Response:** Thank you for your question. We would do the following clarifications. Confidence intervals were obtained using bootstrap resampling. Specifically, simple bootstrap resampling of individuals was performed. In each bootstrap iteration, propensity scores were re-estimated using the same model specification, and stabilized inverse probability of treatment weights (IPTW) were recalculated accordingly. Outcome models were then refitted using the reweighted sample to obtain the bootstrap estimates. We have clarified this procedure in the Methods section of the revised manuscript.

**Reviewer #3:** This paper evaluates the real-world effectiveness of nirmatrelvir in non-hospitalized adults. Utilizing a target trial emulation design, the authors analyze both Long COVID incidence and late-phase clinical outcomes (hospitalization/death) occurring 30 to 180 days post-infection.

*The study is overall well-written and logically organized; nonetheless, I have few minor points that require clarification or refinement.*

We thank the reviewer for the positive evaluation of our study and for the helpful suggestions to improve the clarity and rigor of the manuscript. We have carefully addressed all of the reviewer’s comments and revised the manuscript accordingly. Detailed responses to each comment are provided below.

1) There is a minor inconsistency in the nomenclature used for the intervention throughout the manuscript. While the title mentions only 'Nirmatrelvir', the abstract uses the brand name 'Paxlovid', and the introduction refers to 'nirmatrelvir-ritonavir (Paxlovid)'. Subsequently, 'Paxlovid' is used throughout the text. I recommend the authors standardize the terminology to maintain internal consistency.

**Response:** We have standardized the terminology throughout the manuscript. Specifically, the intervention is now referred to as Paxlovid (or nirmatrelvir/ritonavir) at its first mention, and Paxlovid is used consistently thereafter to maintain internal consistency.

2) Within the 'The PCORNet RECOVER Databases' section, I recommend that the authors provide the full terminology for the abbreviations SDoH (Social Determinants of Health) and NLP (Natural Language Processing) upon their first mention to ensure clarity for all readers.

**Response:** We thank the reviewer for this helpful suggestion. We have now provided the full terminology for the abbreviations Social Determinants of Health (SDoH) and Natural Language Processing (NLP) at their first appearance in the “The PCORNet RECOVER Databases” section of the manuscript.

3) The definition of outcomes and the follow-up period (lines 163–172) are generally well-described. In particular, the use of a competing risk framework for mortality is a robust methodological choice. However, could the authors authors clarify if 'time zero' (line 168) is consistently anchored to the 'index event' (line 139) across all patients? Clarifying whether the 30-to-180-day window is calculated from the date of the first positive test or the date of treatment initiation would help ensure the comparability of the treatment and control groups.

**Response:** We thank the reviewer for this helpful comment and for recognizing the methodological approach used in our study. We clarify that the index date for treated group was defined as the date of the first Paxlovid prescription and the index date for the control group was defined as the date of the first documented evidence of SARS-CoV-2 infection. The follow-up window of 30 to 180 days was from the infection date. We have revised the texts in Method exposure, outcomes sections.

4) I commend the authors for the extensive range of covariates adjusted for in the models. However, while Reference 14 justifies risk factors for severe COVID-19 (lines 181-182), the outcomes of interest here are Long COVID, hospitalization, and mortality. Are there any supporting references that link the selected covariates particularly those that might be related directly to these post-acute outcome to ensure the rationale is consistent ?

**Response:** We thank the reviewer for this helpful suggestion. The selection of baseline covariates was informed not only by previously reported risk factors for severe COVID-19 but also by clinical knowledge regarding factors that may influence post-acute outcomes, including Long COVID, hospitalization, and mortality. In particular, the covariates were selected in consultation with clinicians involved in the RECOVER study to ensure that relevant demographic characteristics and comorbidities were appropriately accounted for in the analyses. We added references and summarized the covariates in Table S2 in the Supplementary Appendix.

5) I suggest the authors briefly define 'negative control outcomes' (falsification endpoints) and explicitly list them in the main text. Providing this explanation would be highly beneficial for clinical readers to understand how these tools are used to detect unmeasured confounding and validate the study's findings.

**Response:** We thank the reviewer for this helpful suggestion. We have added a brief explanation of negative control outcomes (falsification endpoints) in the Methods section to clarify their purpose in assessing potential unmeasured confounding. “We further conducted two negative outcome control studies to explore the possibility of residual confounding.19 Negative control outcomes were selected based on the absence of a plausible causal relationship with Paxlovid treatment and but share the same potential confounders as the primary outcomes. These outcomes were identified based on clinical knowledge and prior literature (e.g., benign neoplasms, skin cancer, etc. as shown in Fig S3-4 in Appendix). If the estimated associations between treatment and these negative control outcomes were close to the null, this would provide evidence supporting the validity of the study design.”

6) The more pronounced HR reduction for mortality compared to hospitalization is consistent with the drug’s mechanism of action and has been observed in other high-risk cohorts, such as the VA study (Xie et al., 2023). This reinforces the plausibility of the results.

Xie et al., JAMA Intern Med 2023:

post–acute death (HR, 0.53; 95% CI, 0.46-0.61)

post–acute hospitalization (HR, 0.76; 95% CI, 0.73-0.80)

vs.

all-cause death (HR, 0.53, 95% CI 0.46 to 0.60)

hospitalization (HR, 0.70, 95% CI 0.68 to 0.73)

**Response:** We thank the reviewer for highlighting this important comparison. We agree that the observed pattern showing a stronger reduction in mortality compared with hospitalization is consistent with findings reported in prior studies, including the VA cohort study by Xie et al. (2023). We also built VA-like cohort and got results in Fig S6 and S7 as sensitivity analyses. We discuss these in the Discussion section to further contextualize and support the plausibility of our findings.

7) I also recommend the authors address the lack of significant benefit observed in low-risk populations in the discussion.

**Response:** We have added a discussion addressing the lack of statistically significant benefit observed among low-risk populations. In particular, we note that individuals at lower baseline risk may experience smaller absolute benefits from antiviral therapy, which could reduce the statistical power to detect differences in these groups. This clarification has been added to the Discussion section of the revised manuscript.

8) Regarding study limitations, the authors should acknowledge that the reliance on ICD codes and EHR data for defining variables and outcomes may introduce misclassification bias.

**Response:** We thank the reviewer for this important suggestion. We agree that the use of ICD codes and electronic health record (EHR) data to define exposures, covariates, and outcomes may introduce potential misclassification. We have now acknowledged this limitation in the Discussion section of the revised manuscript.
