# Supplementary material for: Real-world effectiveness of Paxlovid in protecting Long COVID for outpatient adult patients: A target trial emulation study from the RECOVER Initiative
Source: PLOS Glob Public Health. 2026 Oct 28;6(10):e0005771. doi: 10.1371/journal.pgph.0005771 (PMC13716174; doi:10.1371/journal.pgph.0005771)
Supplement: Peer review history

# Author comment: Author response to Decision Letter 0

**DOI:** 10.1371/journal.pgph.0005771.r001

**Transfer Alert**

This paper was transferred from another journal. As a result, its full editorial history (including decision letters, peer reviews and author responses) may not be present.

15 Dec 2025

# Peer review: Decision Letter 0

**DOI:** 10.1371/journal.pgph.0005771.r002

**Authors:** Ilse Bloom

23 Feb 2026

PGPH-D-25-03662

Real World Effectiveness of Nirmatrelvir in Protecting Long COVID for Outpatient Adult Patients: A Large Scale Observational Cohort Study from the RECOVER Initiative

PLOS Global Public Health

Dear Dr. Zang,

Thank you for submitting your manuscript to PLOS Global Public Health. After careful consideration, we feel that it has merit but does not fully meet PLOS Global Public Health’s publication criteria as it currently stands. Therefore, we invite you to submit a revised version of the manuscript that addresses the points raised during the review process.

The manuscript has been evaluated by three reviewers, and their comments are available below.

Could you please carefully revise the manuscript to address all comments raised?

Please submit your revised manuscript by Apr 09 2026 11:59PM. If you will need more time than this to complete your revisions, please reply to this message or contact the journal office at globalpubhealth@plos.org. When you're ready to submit your revision, log on to https://www.editorialmanager.com/pgph/ and select the 'Submissions Needing Revision' folder to locate your manuscript file.

Please include the following items when submitting your revised manuscript:

- A letter that responds to each point raised by the editor and reviewer(s). You should upload this letter as a separate file labeled 'Response to Reviewers'.
- A marked-up copy of your manuscript that highlights changes made to the original version. You should upload this as a separate file labeled 'Revised Manuscript with Track Changes'.
- An unmarked version of your revised paper without tracked changes. You should upload this as a separate file labeled 'Manuscript'.

Guidelines for resubmitting your figure files are available below the reviewer comments at the end of this letter.

We look forward to receiving your revised manuscript.

Kind regards,

Ilse Bloom

Staff Editor

PLOS Global Public Health

Journal Requirements:

1. We ask that a manuscript source file is provided at Revision. Please upload your manuscript file as a .doc, .docx, .rtf or .tex.

2. We do not publish any copyright or trademark symbols that usually accompany proprietary names, eg (R), (C), or TM  (e.g. next to drug or reagent names). Please remove all instances of trademark/copyright symbols throughout the text, including ® on page 8.

3. Please upload a copy of Figure 1,2 & 3 which you refer to in your text on pages 10,11 & 12. Or, if the figure is no longer to be included as part of the submission please remove all reference to it within the text.

If the reviewer comments include a recommendation to cite specific previously published works, please review and evaluate these publications to determine whether they are relevant and should be cited. There is no requirement to cite these works unless the editor has indicated otherwise.

Additional Editor Comments (if provided):

[Note: HTML markup is below. Please do not edit.]

Reviewers' comments:

Reviewer's Responses to Questions

**Comments to the Author**

1. Does this manuscript meet PLOS Global Public Health’s publication criteria?

Reviewer #1: Partly

Reviewer #2: Yes

Reviewer #3: Yes

**********

2. Has the statistical analysis been performed appropriately and rigorously?

Reviewer #1: Yes

Reviewer #2: Yes

Reviewer #3: Yes

**********

3. Have the authors made all data underlying the findings in their manuscript fully available (please refer to the Data Availability Statement at the start of the manuscript PDF file)?

The PLOS Data policy

Reviewer #1: Yes

Reviewer #2: No

Reviewer #3: Yes

**********

4. Is the manuscript presented in an intelligible fashion and written in standard English?

Reviewer #1: Yes

Reviewer #2: Yes

Reviewer #3: Yes

**********

Reviewer #1: Thank you for the opportunity to review the article.

General comments

Recommend following the TARGET Checklist of Recommended Items to Address in Reports of Studies Emulating a Target Trial when reporting the study (https://jamanetwork.com/journals/jama/fullarticle/2837724).

Recommend consistent use of terminology (e.g. nirmatrelvir vs Paxlovid).

Introduction:

Add a clear rationale for emulating a target trial using the available data for this study.

Consider citing the randomized trial(s) that informed the design of the target trial, if available.

Methods:

Clarify how treatment assignment was determined.

Report the frequency of missing data for all variables, stratified by treatment strategy where applicable.

Inconsistency in study period: Line 144 states March 1, 2022 to February 28, 2023, whereas Figure 1 shows March 1, 2022 to February 1, 2023.

Table 1: consider using “control group” instead of “no treated SARS-CoV-2 Infected.”

Figure 3 presents two infection-time groups, while Table 1 shows three different index times. Consider conducting subgroup analyses based on the three index-time categories defined in Table 1.

Supplementary tables are not included in the submission. Please upload all supplementary tables.

Reviewer #2: How was the index date defined?

Provide a clear definition of censoring.

Were standardized mean differences (SMDs) used to assess balance before weighting, after weighting, or both?

How about categorical variables?

Did the Cox survival model account for competing risks?

Was the proportional hazards assumption tested?

How was the regularization parameter (λ) selected?

Was class imbalance taken into account during model fitting?

What variables were in “all baseline covariates”? Were any variables excluded due to collinearity or missing?

For subgroup analyses, were interaction used or stratified estimation?

Provide details regarding the negative outcome control analyses, including the selection and validation of control outcomes.

How were missing covariate data handled?

More details are needed on the bootstrapping procedure: Was stratified bootstrapping or simple resampling used?

Were propensity scores re-estimated within each bootstrap iteration? Were IPTW weights recalculated in each iteration?

Reviewer #3: This paper evaluates the real-world effectiveness of nirmatrelvir in non-hospitalized adults. Utilizing a target trial emulation design, the authors analyze both Long COVID incidence and late-phase clinical outcomes (hospitalization/death) occurring 30 to 180 days post-infection.

The study is overall well-written and logically organized; nonetheless, I have few minor points that require clarification or refinement.

1) There is a minor inconsistency in the nomenclature used for the intervention throughout the manuscript. While the title mentions only 'Nirmatrelvir', the abstract uses the brand name 'Paxlovid', and the introduction refers to 'nirmatrelvir-ritonavir (Paxlovid)'. Subsequently, 'Paxlovid' is used throughout the text. I recommend the authors standardize the terminology to maintain internal consistency.

2) Within the 'The PCORNet RECOVER Databases' section, I recommend that the authors provide the full terminology for the abbreviations SDoH (Social Determinants of Health) and NLP (Natural Language Processing) upon their first mention to ensure clarity for all readers.

3) The definition of outcomes and the follow-up period (lines 163–172) are generally well-described. In particular, the use of a competing risk framework for mortality is a robust methodological choice. However, could the authors authors clarify if 'time zero' (line 168) is consistently anchored to the 'index event' (line 139) across all patients? Clarifying whether the 30-to-180-day window is calculated from the date of the first positive test or the date of treatment initiation would help ensure the comparability of the treatment and control groups.

4) I commend the authors for the extensive range of covariates adjusted for in the models. However, while Reference 14 justifies risk factors for severe COVID-19 (lines 181-182), the outcomes of interest here are Long COVID, hospitalization, and mortality. Are there any supporting references that link the selected covariates particularly those that might be related directly to these post-acute outcome to ensure the rationale is consistent ?

5) I suggest the authors briefly define 'negative control outcomes' (falsification endpoints) and explicitly list them in the main text. Providing this explanation would be highly beneficial for clinical readers to understand how these tools are used to detect unmeasured confounding and validate the study's findings.

6) The more pronounced HR reduction for mortality compared to hospitalization is consistent with the drug’s mechanism of action and has been observed in other high-risk cohorts, such as the VA study (Xie et al., 2023). This reinforces the plausibility of the results.

Xie et al., JAMA Intern Med 2023:

post–acute death (HR, 0.53; 95% CI, 0.46-0.61)

post–acute hospitalization (HR, 0.76; 95% CI, 0.73-0.80)

vs.

all-cause death (HR, 0.53, 95% CI 0.46 to 0.60)

hospitalization (HR, 0.70, 95% CI 0.68 to 0.73)

7) I also recommend the authors address the lack of significant benefit observed in low-risk populations in the discussion.

8) Regarding study limitations, the authors should acknowledge that the reliance on ICD codes and EHR data for defining variables and outcomes may introduce misclassification bias.

**********

what does this mean?). If published, this will include your full peer review and any attached files.

**Do you want your identity to be public for this peer review?**  If you choose “no”, your identity will remain anonymous but your review may still be made public.

For information about this choice, including consent withdrawal, please see our Privacy Policy

Reviewer #1: No

Reviewer #2: No

Reviewer #3: No

**********

[NOTE: If reviewer comments were submitted as an attachment file, they will be attached to this email and accessible via the submission site. Please log into your account, locate the manuscript record, and check for the action link "View Attachments". If this link does not appear, there are no attachment files.]

# Author comment: Author response to Decision Letter 1

**DOI:** 10.1371/journal.pgph.0005771.r003

14 Apr 2026

Attachment Submitted filename: Response Letter PLOS Global Public Health.docx

# Peer review: Decision Letter 1

**DOI:** 10.1371/journal.pgph.0005771.r004

**Authors:** Julia Robinson

21 May 2026

Real-World Effectiveness of Paxlovid in Protecting Long COVID for Outpatient Adult Patients: A Target Trial Emulation Study from the RECOVER Initiative

PGPH-D-25-03662R1

Dear Prof Zang,

We are pleased to inform you that your manuscript 'Real-World Effectiveness of Paxlovid in Protecting Long COVID for Outpatient Adult Patients: A Target Trial Emulation Study from the RECOVER Initiative' has been provisionally accepted for publication in PLOS Global Public Health.

Before your manuscript can be formally accepted you will need to complete some formatting changes, which you will receive in a follow up email. A member of our team will be in touch with a set of requests.

Please note that your manuscript will not be scheduled for publication until you have made the required changes, so a swift response is appreciated.

IMPORTANT: The editorial review process is now complete. PLOS will only permit corrections to spelling, formatting or significant scientific errors from this point onwards. Requests for major changes, or any which affect the scientific understanding of your work, will cause delays to the publication date of your manuscript.

If your institution or institutions have a press office, please notify them about your upcoming paper to help maximize its impact. If they'll be preparing press materials, please inform our press team as soon as possible -- no later than 48 hours after receiving the formal acceptance. Your manuscript will remain under strict press embargo until 2 pm Eastern Time on the date of publication. For more information, please contact globalpubhealth@plos.org.

Thank you again for supporting Open Access publishing; we are looking forward to publishing your work in PLOS Global Public Health.

Best regards,

Julia Robinson

Executive Editor

PLOS Global Public Health

***********************************************************

Reviewer Comments (if any, and for reference):

Reviewer's Responses to Questions

**Comments to the Author**

Reviewer #2: All comments have been addressed

Reviewer #3: All comments have been addressed

**********

publication criteria?

Reviewer #2: (No Response)

Reviewer #3: Yes

**********

3. Has the statistical analysis been performed appropriately and rigorously?

Reviewer #2: (No Response)

Reviewer #3: Yes

**********

4. Have the authors made all data underlying the findings in their manuscript fully available (please refer to the Data Availability Statement at the start of the manuscript PDF file)?

The PLOS Data policy

Reviewer #2: (No Response)

Reviewer #3: Yes

**********

5. Is the manuscript presented in an intelligible fashion and written in standard English?

Reviewer #2: (No Response)

Reviewer #3: Yes

**********

Reviewer #2: All my concerns are addressed.

Reviewer #3: I really appreciated the study and the authors’ effort to conduct an observational analysis in the best possible way using a framework compatible with target trial emulation. In addition, the authors have addressed all of my comments. From my perspective, I believe the study is suitable for publication.

**********

what does this mean?). If published, this will include your full peer review and any attached files.

**Do you want your identity to be public for this peer review?**  If you choose “no”, your identity will remain anonymous but your review may still be made public.

For information about this choice, including consent withdrawal, please see our Privacy Policy

Reviewer #2: No

Reviewer #3: No

**********
